# Almost minimum error discrimination of *N*-ary weak coherent states by Jaynes-Cummings Hamiltonian dynamics

**DOI:** 10.1038/s41598-019-55589-7

**Published:** 2019-12-23

**Authors:** Min Namkung, Younghun Kwon

**Affiliations:** Department of Applied Physics, Hanyang University, Ansan, Kyounggi-Do 425-791 South Korea

**Keywords:** Quantum information, Quantum mechanics

## Abstract

Quantum state discrimination of coherent states has been one of important problems in quantum information processing. Recently, R. Han *et al*. showed that minimum error discrimination of two coherent states can be nearly done by using Jaynes-Cummings Hamiltonian. In this paper, based on the result of R. Han *et al*., we propose the methods where minimum error discrimination of more than two weak coherent states can be nearly performed. Specially, we construct models which can do almost minimum error discrimination of three and four coherent states. Our result can be applied to quantum information processing of various coherent states.

## Introduction

Quantum system comprising of non-orthogonal quantum states cannot be perfectly discriminated. The property is at the heart of quantum physics. Therefore, there has been extensive researches in quantum state discrimination. The quantum state discrimination can be understood as a game where a sender Alice and a receiver Bob participate in. In the game, with a prior probability, Alice prepares a quantum state out of *N* quantum states and sends it to Bob. Then, Bob sets up a measurement to discriminate the quantum state of Alice. The method for Bob to establish a measurement depends on the strategy that Bob chooses since Bob’s result can be conclusive or inconclusive. When Bob is allowed to get only conclusive results, the best strategy is to make the error probability of conclusive results minimally. The strategy is called minimum error discrimiation^1–6^. Meanwhile, when the inconclusive result is allowed, there is a strategy to discriminate Alice’s quantum state without an error. It is called unambiguous discrimination, where the failure probability should be minimized^7–12^. In fact, there are other strategies-maximal confidence strategy^13^, error margin strategy^14–17^, and fixed error rate strategy^18–21^. And sequential strategy has been recently introduced^22–29^.

Quantum state discrimination can be applied to quantum key distribution (QKD)^30^, quantum random number generation^31^, and quantum state tomography^32^. Especially, since a coherent state is robust to noise, minimum error discrimination of coherent states can provide realistic quantum communication^33^. Therefore, one needs to construct an optical receiver for performing minimum error discrimination of coherent states. The major obstacle in minimum error discrimination of coherent states is to implement a projective measurement which can provide the Helstrom bound since the implementation of a projective measurement is known to be extremely difficult^34^. In 1973, Dolinar^35^ proposed a receiver to discriminate two coherent states with minimum error. Since then, by extending the idea of Dolinar, receivers to discriminate *N* coherent states have been proposed^36–45^. However, it is recently proved that when *N* > 2, Dolinar-type receiver cannot perform minimum error discrimination^46^.

Therefore, one needs to consider a different type of measurement. The possible idea is to use Neumark formalism (which contains an auxiliary system and global unitary operator). Recently, R. Han *et al*.^47,48^ showed that Neumark formalism in minimum error discrimination of two coherent states could almost reach the Helstrom bound. The similar idea was also discussed by M. P. da Silva^49^. M. P. da Silva uses a quantum computer to construct a global unitary operator, but R. Han *et al*. uses a Jaynes-Cummings Hamiltonian^50^ which can be experimentally implemented^51–53^.

In this report, we consider minimum error discrimination of multiple (more than two) coherent states. Based on the argument of R. Han *et al*.^47,54^, we use *N*−level atom to perform conclusive discrimination of *N* coherent states. When *N* > 2, there exist various interaction Hamiltonians between coherent states and an atom^55–57^. For example, when *N* = 3, interaction Hamiltonian between coherent states and an atom can be described by ladder configuration, lambda (Λ) configuration, and vee (V) configuration. We show that by using three-level atom of ladder configuration, the error bound of minimum error discrimination for three-phase shift keying (PSK) signals or three amplitude shift keying (ASK) signals with identical prior probabilities can nearly reach the Helstrom bound. Further, in terms of the extracted information, we explain how the quantum state discrimination strategy using the ladder configuration can nearly reach the Helstrom bound^48^. It is because the extracted information from the measurement strategy based on the ladder configuration reaches nearly one. It implies that Bob can extract almost every information of Alice’s quantum state by measuring the atom. When *N* = 4, there are more models than those of *N* = 3. We prove that by applying Tavis-Cummings Hamiltonian, which has an interaction between coherent states and two-level atoms, the Helstrom bound can be nearly obtained in minimum error discrimination of four PSK signals with identical prior probabilities^58,59^.

## Results

### Light-atom interaction hamiltonian

Recently, it was shown that the minimum error discrimination could be performed by Neumark formalism, instead of projective measurement. The measurement apparatus is composed of an auxiliary system and a global unitary operation. When in a prior probability Alice prepares and sends one of *N* quantum states to Bob, Bob constructs an auxiliary system with *N* dimensional Hilbert space and performs a global unitary operator on Alice’s quantum state and an auxiliary system. Then, Bob carries out a projective measurement on the auxiliary system, which always provides a conclusive result. What Bob has to do is to construct suitable global unitary operator and projective measurement, to minimize the error probability.

The Neumark formalism can be useful in discrimination of coherent states. It is because it is difficult to implement the projective measurement, which can discriminate coherent states with a minimum error, by a beam splitter, displacement operation, and electric feedback. As mentioned before, Jaynes-Cummings Hamiltonian and auxiliary system of a two-level atom can be used for discrimination of two coherent states. R. Han *et al*. showed that Jaynes-Cummings Hamiltonian could discriminate two coherent states with almost minimum error. From the result of R. Han *et al*., it is natural to ask whether *N* coherent states can be discriminated by a unitary operation and a measurement. If possible, one should find the Hamiltonian which can perform the discrimination. In this report, we will provide the answer to it.

**Figure 1:**
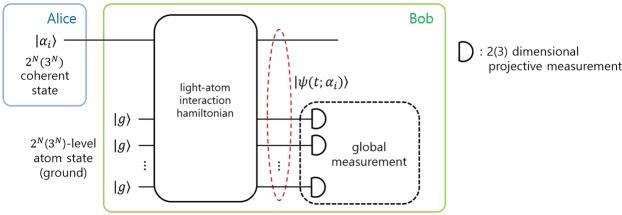
Design for minimum error discrimination of 2^*N*^ (3^*N*^) coherent states. In this design the global measurement consists of *N* number of 2(3) dimensional projective measurement.

**Figure 2:**
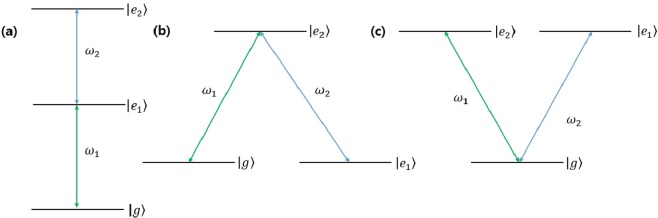
Interaction model between light and three-level atom. (**a**–**c**) Denote ladder configuration, Λ configuration, and V configuration, respectively. Here, |*g*〉, |*e*_1_〉, and |*e*_2_〉 are the ground state, the 1st excited state, and the 2nd excited state of a three-level atom. *ω*_1_ and *ω*_2_ are light frequencies. Because we consider minimum error discrimination of a single-shot coherent state, we use a ladder configuration with identical *ω*_1_ and *ω*_2_. Therefore, in Λ(V) configuration, |*g*〉 and |*e*_1_〉(|*e*_1_〉 and |*e*_2_〉) are degenerate.

In general, for the discrimination of *N* coherent states, *N*−level atom can be used as an auxiliary system. In this report, we deal with the special cases such as *N* = 3^*m*^ (among the odd case) and *N* = 2^*m*^ (among the even case) (see Fig. 1). The simplest case of odd one is *N* = 3. In this case, the three-level atom is needed for the auxiliary system. The basic structure of three-level atom can be found in Fig. 2: “ladder” configuration, “lambda (Λ)” configuration and “Vee (V)” configuration. In the “ladder” configuration (Fig. 2(a)), the transition between atomic levels is only allowed between the ground state (*g*) and the 1st excited state (|*e*_1_〉) or between the 1st excited state (|*e*_1_〉) and the 2nd excited state (|*e*_2_〉). In “lambda (Λ)” configuration (Fig. 2(b)), the transition between atomic levels is only allowed between the ground state and the 2nd excited state or between the 1st excited state and the 2nd excited state. In “Vee (V)” configuration (Fig. 2(c)), the transition between atomic levels is only allowed between the ground state and the 2nd excited state or between the ground state and the 1st excited state. Here, we consider only a single-shot discrimination. And in Λ(V) configuration, |*g*〉 and |*e*_1_〉 (|*e*_1_〉 and |*e*_2_〉) are degenerate. The Hamiltonians of three configurations can be written as

1 $$\begin{array}{ccc}{\hat{H}}_{I}^{(L)}({\rm{t}}) & = & \hslash g(t)\{{w}_{1}|{e}_{1}\rangle \langle g|\otimes \hat{a}+{w}_{2}|{e}_{2}\rangle \langle {e}_{1}|\otimes \hat{a}\}+\hslash g(t)\{{w}_{1}^{\ast }|g\rangle \langle {e}_{1}|\otimes {\hat{a}}^{\dagger }+{w}_{2}^{\ast }|{e}_{1}\rangle \langle {e}_{2}|\otimes {\hat{a}}^{\dagger }\},\\ {\hat{H}}_{I}^{(\varLambda )}(t) & = & \hslash g(t)\{{w}_{1}|{e}_{2}\rangle \langle g|\otimes \hat{a}+{w}_{2}|{e}_{2}\rangle \langle {e}_{1}|\otimes \hat{a}\}+\hslash g(t)\{{w}_{1}^{\ast }|g\rangle \langle {e}_{2}|\otimes {\hat{a}}^{\dagger }+{w}_{2}^{\ast }|{e}_{1}\rangle \langle {e}_{2}|\otimes {\hat{a}}^{\dagger }\},\\ {\hat{H}}_{I}^{({\rm{V}})}(t) & = & \hslash g(t)\{{w}_{1}|{e}_{2}\rangle \langle g|\otimes \hat{a}+{w}_{2}|{e}_{1}\rangle \langle g|\otimes \hat{a}\}+\hslash g(t)\{{w}_{1}^{\ast }|g\rangle \langle {e}_{2}|\otimes {\hat{a}}^{\dagger }+{w}_{2}^{\ast }|g\rangle \langle {e}_{1}|\otimes {\hat{a}}^{\dagger }\mathrm{\}}.\end{array}$$

Here, *g* (*t*) is a real function. And $\hat{a}$ (${\hat{a}}^{\dagger }$) is an annihilation (creation) operator, satisfying $\hat{a}|n\rangle =\sqrt{n}|n-1\rangle$ and ${\hat{a}}^{\dagger }|n\rangle =\sqrt{n+1}|n+1\rangle$, where |*n*〉 represents a Fock basis. Here, *w*_1_ and *w*_2_ are transition process weights. The lower index of transition process weight has the following meanings. In the ladder configuration, *w*_1_(*w*_2_) denote the transition weight between the ground state and the 1st excited state (between the 1st excited state and the 2nd excited state). In the Λ configuration, *w*_1_(*w*_2_) is the transition weight between the ground state and the 2nd excited state (between the 1st excited state and the 2nd excited state). In V configuration, *w*_1_(*w*_2_) denotes the transition weight between the ground state and the 2nd excited state (between the ground state and the 1st excited state). The transition process weight is determined by the coupling strength between light and atom. For example, in the ladder configuration, when *w*_1_ > *w*_2_, light strongly interacts with an atom in the ground state or an atom in the 1st excited state. If *w*_1_ < *w*_2_, light strongly interacts with an atom in the 1st excited state or with an atom in the 2nd excited state. Generally, coupling strengths have different values according to the level where a transition occurs^60^.

If frequencies of two light are not equal in Fig. 2, the ladder configuration needs two coherent light with different frequencies. Because we consider minimum error discrimination of a single-shot coherent state, we use the ladder configuration with an identical light frequency. Because Λ configuration and V configuration need two coherent lights with different polarizations, ladder configuration is more suitable in minimum error discrimination of a single-shot coherent state than two other configurations. (The detailed argument is given in the next Section).

In the case of *N* = *even*, there are various configurations of atomic states. In the case of *N* = 4, we may take the interaction between two two-level atoms and four coherent states. The interaction Hamiltonian is the following Tavis-Cummings Hamiltonian:

2 $$\begin{array}{rcl}{\hat{H}}_{I}^{(TC)} & = & \hslash g(t)\{{w}^{(1)}|e\rangle \langle g|\otimes \hat{I}\otimes \hat{a}+{w}^{(2)}\hat{I}\otimes |e\rangle \langle g|\otimes \hat{a}\}\\  &  & +\hslash g(t)\{{w}^{(1)\ast }|g\rangle \langle e|\otimes \hat{I}\otimes {\hat{a}}^{\dagger }+{w}^{(2)\ast }\hat{I}\otimes |g\rangle \langle e|\otimes {\hat{a}}^{\dagger }\}.\end{array}$$

Here, *w*^(1)^ (*w*^(2)^) is the transition weight between the ground state of the first (the second) atom and the excited state of the first (the second) atom. In the case of *N* = 2^*m*^ or *N* = 3^*m*^, one can construct a Jaynes-Cummings type Hamiltonian according to the following rules: When a photon comprising coherent state disappears, the energy level of the atom goes one step up. Similarly, when a photon comprising coherent state adds up, the energy level of the atom goes one step down. To discriminate *N* = 2^*m*^ coherent states, one can construct the following Jaynes-Cummings Hamiltonian:

3 $${\hat{H}}_{I}^{(n)}=\hslash g(t)\mathop{\sum }\limits_{k=0}^{n-1}\,[{w}^{(k+1)}({\hat{I}}^{\otimes k})\otimes |e\rangle \langle g|\otimes ({\hat{I}}^{\otimes (n-k-1)})\otimes \hat{a}+{w}^{(k+1)\ast }({\hat{I}}^{\otimes k})\otimes |g\rangle \langle e|\otimes ({\hat{I}}^{\otimes (n-k-1)})\otimes {\hat{a}}^{\dagger }].$$

Here, *w*^(*k*+1)^ is the transition process weight, where the upper index denotes the label of atom in a transition. When *n* = 1, Eq. (3) is identical to Jaynes-Cummings Hamiltonian. When *n* = 2, Eq. (3) becomes Tavis-Cummings Hamiltonian.

When one discriminates *N* = 3^*m*^ coherent states, the interaction Hamiltonian can be constructed by the following atomic configuration:

4 $$\begin{array}{ccc}{\hat{H}}_{I}^{(n,L)} & = & \hslash g(t)\mathop{\sum }\limits_{k\mathrm{=0}}^{n-1}\,[({\hat{I}}^{\otimes k})\otimes \{{w}_{1}^{(k+\mathrm{1)}}|{e}_{1}\rangle \langle g|+{w}_{2}^{(k+\mathrm{1)}}|{e}_{2}\rangle \langle {e}_{1}|\}\otimes ({\hat{I}}^{\otimes (n-k-\mathrm{1)}})\otimes \hat{a}+H\mathrm{}.c\mathrm{}.],\\ {\hat{H}}_{I}^{(n,\varLambda )} & = & \hslash g(t)\mathop{\sum }\limits_{k\mathrm{=0}}^{n-1}\,[({\hat{I}}^{\otimes k})\otimes \{{w}_{1}^{(k+\mathrm{1)}}|{e}_{2}\rangle \langle g|+{w}_{2}^{(k+\mathrm{1)}}|{e}_{2}\rangle \langle {e}_{1}|\}\otimes ({\hat{I}}^{\otimes (n-k-\mathrm{1)}})\otimes \hat{a}+H\mathrm{}.c\mathrm{}.],\\ {\hat{H}}_{I}^{(n,{\rm{V}})} & = & \hslash g(t)\mathop{\sum }\limits_{k\mathrm{=0}}^{n-1}\,[({\hat{I}}^{\otimes k})\otimes \{{w}_{1}^{(k+\mathrm{1)}}|{e}_{2}\rangle \langle g|+{w}_{2}^{(k+\mathrm{1)}}|{e}_{1}\rangle \langle g|\}\otimes ({\hat{I}}^{\otimes (n-k-\mathrm{1)}})\otimes \hat{a}+H\mathrm{}.c\mathrm{}.].\end{array}$$

${\hat{H}}_{I}^{(n,L)}$, ${\hat{H}}_{I}^{(n,\varLambda )}$, and ${\hat{H}}_{I}^{(n,{\rm{V}})}$ are Ladder, Λ, and V configuration, respectively. In Eqs. (1) and (2), ${w}_{i}^{(k)}$ can be thought as a weight of transition process. And ${w}_{i}^{(k)}$(*i* ∈ {1, 2}) is a transition process weight of *k*-th atom. The upper index *i* has the following meaning. In the ladder configuration, ${w}_{1}^{(k)}$ (${w}_{2}^{(k)}$) denotes the transition weight between the ground state and the 1st excited state of *k*−th atom (between the 1st excited state and the 2nd excited state of *k*−th atom). In Λ configuration, ${w}_{1}^{(k)}$ (${w}_{2}^{(k)}$) is the transition weight between the ground state and the 2nd excited state of *k*−th atom (between the 1st excited state and the 2nd excited state of *k*−th atom. In V configuration, ${w}_{1}^{(k)}$ (${w}_{2}^{(k)}$) denotes the transition weight between the ground state and the 1st excited state of *k*−th atom (between the ground state and the 2nd excited state of of *k*−th atom). Since Eqs. (3) and (4) are complicated, we do not deal in detail with the dynamics of Eqs. (3) and (4). Now, let us suppose that |*ψ*(*t*)〉 is a light-atom state after a time *t*. The light-atom state |*ψ*(*t*)〉 should satisfy the following time-dependent Schrodinger equation:

5 $$i\hslash \frac{\partial }{\partial t}|\psi (t;\alpha )\rangle ={\hat{H}}_{I}|\psi (t;\alpha )\rangle ,$$

where ${\hat{H}}_{I}$ is an interaction Hamiltonian. When *N* = 3, ${\hat{H}}_{I}\in \{{\hat{H}}_{I}^{(L)},{\hat{H}}_{I}^{(\Lambda )},{\hat{H}}_{I}^{({\rm{V}})}\}$. And the initial light-atom state is given by |*ψ*(0; *α)*〉 = |*g*〉 ⊗ |*α*〉. When *N* = 4, ${\hat{H}}_{I}$ becomes Tavis-Cummings Hamiltonian ${\hat{H}}_{I}^{(TC)}$. In this case, an initial light-atom state is given by |*ψ*(0; *α*)〉 = *|g*〉 ⊗ |*g*〉 ⊗ |*α*〉. In addition, when one discriminates 2^*N*^ coherent states, an initial light-atom state becomes *ψ*(0;*α*) *=* |*g*〉^⊗*N*^ ⊗ |*α*〉.

#### Three coherent states case

First, let us consider the interaction between coherent state and three-level atom. Suppose that Bob’s quantum state of a three-level atom is initially in the ground state. Then, the initial state of light-atom becomes *ψ*(0;*α*) = |*g*〉 ⊗ |*α*〉, where |*α*〉 represents a coherent state. After an interaction, the light-atom state is described by

6 $$|\psi (t;\alpha )\rangle =\mathop{\sum }\limits_{n\mathrm{=0}}^{\infty }\,\{{c}_{g,n}(\Phi (t);\alpha )|g\rangle \otimes |n\rangle +{c}_{{e}_{1},n}(\Phi (t);\alpha )|{e}_{1}\rangle \otimes |n\rangle +{c}_{{e}_{2},n}(\Phi (t);\alpha )|{e}_{2}\rangle \otimes |n\rangle \},$$

where Φ(*t*) is a function defined by

7 $$\Phi (t)={\int }_{0}^{t}\,d\tau g(\tau \mathrm{)}.$$

Therefore, if *t* = 0, we have Φ(*t*) = 0. Since the coefficient of Eq. (7) is a function of Φ, time-evolution of light-atom state does not severely depend on the specific form of *g*(*t*). When a ladder configuration is applied to coherent states and a three-level atom, the coefficient of Eq. (6) are analytically obtained as

8 $$\begin{array}{rcl}{c}_{g,n}(\Phi ;\alpha ) & = & \frac{1}{{f}_{n}^{2}}[|{w}_{1}{|}^{2}n{\alpha }_{n}\,\cos \,\{{f}_{n}\Phi \}+|{w}_{2}{|}^{2}(n-\mathrm{1)}{\alpha }_{n}],\\ {c}_{{e}_{1},n-1}(\Phi ;\alpha ) & = & -\frac{i}{{f}_{n}}{w}_{1}\sqrt{n}{\alpha }_{n}\,\sin \,\{{f}_{n}\Phi \},\\ {c}_{{e}_{2},n-2}(\Phi ;\alpha ) & = & \frac{1}{{f}_{n}^{2}}{w}_{1}{w}_{2}\sqrt{n(n-\mathrm{1)}}[{\alpha }_{n}\,\cos \,\{{f}_{n}\Phi \}-{\alpha }_{n}\mathrm{]}.\end{array}$$

Here, ${\alpha }_{n}={e}^{-\frac{|\alpha {|}^{2}}{2}}\frac{{\alpha }^{n}}{n!}$ and ${f}_{n}=\sqrt{|{w}_{1}{|}^{2}n+|{w}_{2}{|}^{2}(n-\mathrm{1)}}$. Since *f*_*n*_ ≥ 0 for every *n*, the inequality condition |*w*_1_| ≥ |*w*_2_| should be satisfied. If a Λ configuration is applied to coherent states and a three-level atom, the coefficient of Eq. (6) are analytically obtained as

9 $$\begin{array}{rcl}{c}_{g,n}(\Phi ;\alpha ) & = & \frac{|{w}_{1}{|}^{2}{\alpha }_{n}}{{\Omega }^{2}}\,\cos \,\{\sqrt{n}\Omega \Phi \}+\frac{|{w}_{2}{|}^{2}}{{\Omega }^{2}}{\alpha }_{n},\\ {c}_{{e}_{1},n}(\Phi ;\alpha ) & = & \frac{{w}_{1}^{\ast }{w}_{2}}{{\Omega }^{2}}[\,\cos \,\{\sqrt{n}\Omega \Phi \}-\mathrm{1],}\\ {c}_{{e}_{2},n-1}(\Phi ;\alpha ) & = & -\,i\frac{{w}_{1}^{\ast }}{\Omega }{\alpha }_{n}\,\sin \,\{\sqrt{n}\Omega \Phi \mathrm{\}}.\end{array}$$

Here, $\Omega =\sqrt{|{w}_{1}{|}^{2}+|{w}_{2}{|}^{2}}$. When a V configuration is applied to coherent states and a three-level atom, the coefficient of Eq. (6) are analytically obtained as

10 $$\begin{array}{rcl}{c}_{g,n}(\Phi ;\alpha ) & = & {\alpha }_{n}\,\cos \,\{\sqrt{n}\Phi \},\\ {c}_{{e}_{1},n-1}(\Phi ;\alpha ) & = & -\,i\frac{{w}_{2}^{\ast }}{\Omega }{\alpha }_{n}\,\sin \,\{\sqrt{n}\Omega \Phi \},\\ {c}_{{e}_{2},n-1}(\Phi ;\alpha ) & = & -\,i\frac{{w}_{1}^{\ast }}{\Omega }{\alpha }_{n}\,\sin \,\{\sqrt{n}\Omega \Phi \mathrm{\}}.\end{array}$$

A detailed derivation from Eqs. (8) to (10) can be found in the Method section.

#### Four coherent states case

Now, let us consider the discrimination of four coherent states. For the case, we use an interaction between coherent states and two two-level atoms. Assuming that every initial quantum state of two two-level atoms is in the ground state, the light-atom state after an interaction is described by

11 $$\begin{array}{rcl}\psi (t;\alpha ) & = & \mathop{\sum }\limits_{n=0}^{\infty }\,\{{c}_{g,g,n}(\Phi (t),\alpha )g\otimes g\otimes n+{c}_{g,e,n}(\Phi (t),\alpha )g\otimes e\otimes n\\  &  & +{c}_{e,g,n}(\Phi (t),\alpha )e\otimes g\otimes n+{c}_{e,e,n}(\Phi (t),\alpha )e\otimes e\otimes n\mathrm{\}}.\end{array}$$

Then, the coefficients of Eq. (11) are given by

12 $$\begin{array}{rcl}{c}_{g,g,n}(\Phi ,\alpha ) & = & \frac{n-1}{2n-1}{\alpha }_{n}+\frac{n}{2n-1}{\alpha }_{n}\,\cos \,\{\sqrt{\mathrm{2(2}n-\mathrm{1)}}\Phi \},\\ {c}_{g,e,n-1}(\Phi ,\alpha ) & = & -\,i\frac{\sqrt{n}}{\sqrt{\mathrm{2(2}n-\mathrm{1)}}}{\alpha }_{n}\,\sin \,\{\sqrt{\mathrm{2(2}n-\mathrm{1)}\Phi }\},\\ {c}_{e,g,n-1}(\Phi ,\alpha ) & = & -\,i\frac{\sqrt{n}}{\sqrt{\mathrm{2(2}n-\mathrm{1)}}}{\alpha }_{n}\,\sin \,\{\sqrt{\mathrm{2(2}n-\mathrm{1)}\Phi }\},\\ {c}_{e,e,n-2}(\Phi ,\alpha ) & = & \frac{\sqrt{n(n-\mathrm{1)}}}{2n-1}{\alpha }_{n}\,\cos \,\{\sqrt{\mathrm{2(2}n-\mathrm{1)}}\Phi \}-\frac{\sqrt{n(n-\mathrm{1)}}}{2n-1}{\alpha }_{n}\mathrm{}.\end{array}$$

The form of Eq. (12) is similar to the coefficients of ladder configuration with *w*_1_ = *w*_2_, which implies that Tavis-Cumming Hamiltonian is similar to ladder configuration. In Eq. (12), *c*_*g*,*e*,*n*−1_(Φ, *α*) and *c*_*e*,*g*,*n*−1_(Φ, *α*) are equivalent. Therefore, the time-evolution of Eq. (12) is symmetric in the interchanging of two two-level atoms.

Symmetric Tavis-Cummings type interaction is equivalent to three-level ladder configuration with symmetric coupling (*w*_1_ = *w*_2_). When a non-symmetric coupling is considered, Tavis-Cummings type interaction may provide more general structure of quantum measurement.

### Ternary coherent states discrimination

**Figure 3:**
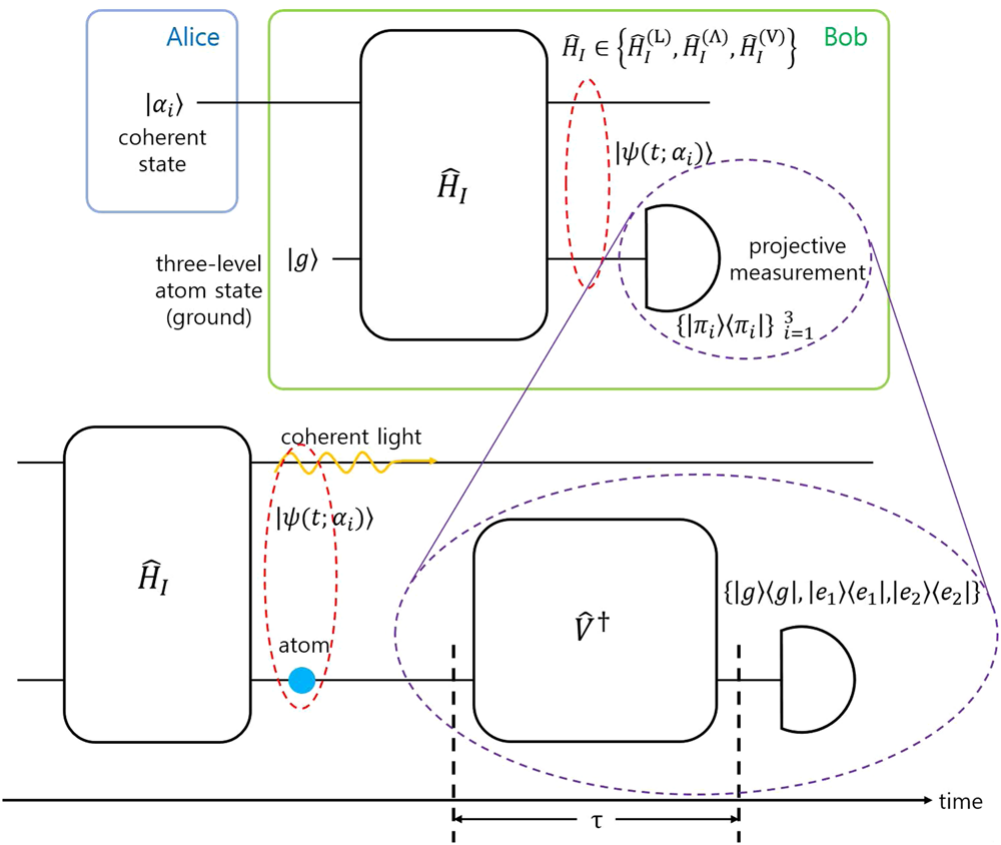
Minimum error discrimination based on Neumark formalism. Alice prepares a quantum state with a prior probability, out of {|*α*_1_〉, |*α*_2_〉, |*α*_3_〉}. Bob performs an interaction between Alice’s coherent state and the ground state of a three-level atom. The interaction Hamiltonian is described by Ladder, Λ, and V configuration. After the interaction, Bob performs a projective measurement on the atomic part of |*ψ*(*t*; *α*)〉. The figure describes the method to implement a projective measurement. After an interaction between a coherent state and the three-level atom, the atom interacts one more time with an external light. The process can be described by unitary transformation ${\hat{V}}^{\dagger }$. After a time interval of *τ*, a state of the atom is measured on an energy basis.

In this section, we consider the discrimination of three coherent states. Figure 3 displays the process. Suppose that Alice prepares one of three coherent states |*α*_*i*_〉 ∈ {|*α*_1_〉, |*α*_2_〉, |*α*_3_〉}, with a prior probability *q*_*i*_. Bob uses a three-level atom as an auxiliary system, where the atom initially lies in the ground state. When a coherent state |*α*_*i*_〉 interacts with the three-level atom, Bob has a light-atom state |*ψ*(*t*;*α*_*i*_)〉. If Bob performs a projective measurement $\{{\hat{\Pi }}_{1},{\hat{\Pi }}_{2},{\hat{\Pi }}_{3}\}$ = {|*π*_1_〉〈*π*_1_|, |*π*_2_〉〈*π*_2_|, |*π*_3_〉〈*π*_3_|} on the atomic part and obtains *i* as the result of measurement, Bob regards the coherent state as |*α*_*i*_〉. The guessing probability of Bob is given by

13 $$\begin{array}{rcl}{P}_{g}=\,{\rm{\max }}\,{P}_{s} & = & \mathop{{\rm{\max }}}\limits_{{\{{\hat{\varPi }}_{i}\}}_{i=1}^{3},\Phi }\frac{1}{3}\mathop{\sum }\limits_{i=1}^{3}\,{\langle \psi (\Phi ;{\alpha }_{i}){|}_{AL}{\{|{\pi }_{i}\rangle }_{A}\langle {\pi }_{i}|\otimes {\hat{I}}_{L}\}|{\rm{\psi }}(\Phi ;{\alpha }_{i})\rangle }_{AL}\\  & = & \mathop{{\rm{\max }}}\limits_{{\{{\hat{\varPi }}_{i}\}}_{i=1}^{3},\Phi }\frac{1}{3}\mathop{\sum }\limits_{i=1}^{3}\,|(\langle {\pi }_{i}{|}_{A}\otimes {\hat{I}}_{L})|\psi (\Phi ;{\alpha }_{i}){\rangle }_{AL}{|}^{2}\mathrm{}.\end{array}$$

The success probability *P*_*s*_ is optimized by Φ = Φ(*t*), corresponding to the function of interaction time, and the structure of projective measurement. The orthonormal vector *π*_*i*_ composing of projective measurement is obtained by a unitary transformation $V\in {{\mathbb{C}}}^{3\times 3}$ based on the three-level atom:

14 $$[\begin{array}{c}|{\pi }_{1}\rangle \\ |{\pi }_{2}\rangle \\ |{\pi }_{3}\rangle \end{array}]=V[\begin{array}{c}|g\rangle \\ |{e}_{1}\rangle \\ |{e}_{2}\rangle \end{array}],\,V=[\begin{array}{ccc}{v}_{11} & {v}_{12} & {v}_{13}\\ {v}_{21} & {v}_{22} & {v}_{23}\\ {v}_{31} & {v}_{32} & {v}_{33}\end{array}]\mathrm{}.$$

By substituting Eqs. (6) and (14) into Eq. (13), the guessing probability becomes

15 $${P}_{g}=\mathop{max}\limits_{{\{{\hat{\varPi }}_{i}\}}_{i=1}^{3},\Phi }\frac{1}{3}\mathop{\sum }\limits_{i=1}^{3}\,\mathop{\sum }\limits_{n=0}^{\infty }|{v}_{i1}^{\ast }{c}_{g,n}(\Phi ;{\alpha }_{i})+{v}_{i2}^{\ast }{c}_{{e}_{1},n}(\Phi ;{\alpha }_{i})+{v}_{i3}^{\ast }{c}_{{e}_{2},n}(\Phi ;{\alpha }_{i}{)|}^{2}\mathrm{}.$$

*V* satisfies the orthonormal condition ${\sum }_{i=1}^{3}\,{v}_{ij}^{\ast }{v}_{ik}={\delta }_{jk}$^61^. In Eq. (15), the structure of configuration and the interaction weight affect only on coefficients *c*_*g*,*n*_(Φ; *α*_*i*_), *c*_*e*1,*n*_(Φ; *α*_*i*_), *c*_*e*2,*n*_(Φ; *α*_*i*_). In general, *v*_*ij*_ is a complex number, and *V* is composed of eighteen real numbers. By the equality constraint of *V*, the number of the free variable of *V* becomes nine. Now, let us denote *V* as *V* = exp(*iM*), where *M* is a three-dimensional Hermitian matrix.

Since it is difficult to analytically obtain guessing probability Eq. (13), we calculate it numerically. First, we choose a Hermitian matrix *M*. There is no constraint on free variables of *M*, and one can apply the Powell method or Fletcher-Reeve method, which is a non-constrained optimization method, to the problem. Unfortunately, the optimum provided by a numerical method is not usually a global one. Therefore, to search for the global optimum, one should try many iterations.

As a first example, we consider the discrimination of three coherent states {|*α*〉, |*α* exp(2*πi*/3)〉|*α* exp(−2*πi*/3)〉}, prepared by equal prior probabilities. The coherent states are called 3PSK signals^33^. For the discrimination of 3PSK signals, one should use the projective measurement in the Hilbert space spanned by 3PSK signals, to achieve the Helstrom bound. However, the implementation of projective measurement is really difficult. Even though there have been many attempts by Dolinar-type receiver for the Helstrom bound, it was recently proven that Dolinar-type receiver could not achieve the Helstrom bound. Meanwhile, non-projective measurement based on Neumark formalism can discriminate 3PSK signals with almost minimum error. However, Jaynes-Cummings Hamiltonian cannot construct a general Neumark formalism, but we will show that a Hamiltonian formalism may nearly reach the Helstrom bound for the 3PSK signals.

**Figure 4:**
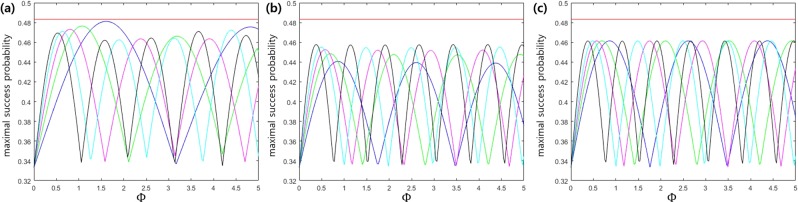
Optimal success probability when the amplitude of coherent state is |*α*| = 0.2. Here, the red solid line denotes the Helstrom bound[1]. (**a**–**c**) Are the cases of ladder configuration, Λ configuration, and V configuration. In (**a**), darker-blue, green, purple, light-blue, black solid lines correspond to the cases of *w*_1_ = 1.0, 1.5, 2.0, 2.5, 3.0, at *w*_2_ = 1.0. In (**b**,**c**), darker-blue, green, purple, light-blue, and black solid lines correspond to the cases of *w*_1_ = 1.5, 2.0, 2.5, 3, and 4, at *w*_2_ = 1.0.

Figure 4 shows the success probability (*P*_*s*_) when the interaction hamitonian is ladder configuration. Here, we use |*α*| = 0.2 and the weights of *w*_1_ ∈ {1, 1.5, 2, 2.5, 3} and *w*_2_ = 1. We found that the Helstrom bound can be nearly achieved when two weights are identical. That is, to come close to Helstrom bound, interaction strength of light and atom between the ground state and 1st excited state needs to be the same as that between 1st excited state and 2nd excited state. Figure 4(b,c) display the success probability (*P*_*s*_) when the interaction hamitonian are Λ configuration and V configuration. In these cases, we use |*α*| = 0.2, *w*_1_ ∈ {2, 2.5, 3, 3.5, 4}, and *w*_2_ = 1. Figure 4(b) shows that Λ configuration provides a maximum of the success probability when *w*_1_ = 4, but Fig. 4(c) shows that in V configuration a maximum of the success probability is independent of weight. The condition of ladder configuration for nearly reaching the Helstrom bound is Φ(*t*) = 1.606. The optimal unitary transformation of projective measurement is given by

16 $${V}_{{\rm{opt}}}=\frac{1}{\sqrt{3}}[\begin{array}{ccc}1 & \exp (i\pi \mathrm{/2)} & \exp (i\pi )\\ 1 & \exp (-i\pi \mathrm{/6)} & \exp (-i\pi \mathrm{/3)}\\ 1 & \exp (-i5\pi \mathrm{/6)} & \exp (i\pi \mathrm{/3)}\end{array}]\mathrm{}.$$

We found the optimal condition numerically. The unitary transformation of Eq. (16) transforms atom states |*g*〉, |*e*_1_〉, |*e*_2_〉 into the following three quantum states:

17 $$\begin{array}{rcl}|g\rangle \to {V}_{{\rm{opt}}}|g\rangle  & = & \frac{1}{\sqrt{3}}(|g\rangle +\exp (i\pi \mathrm{/2)}|{e}_{1}\rangle +\exp (i\pi )|{e}_{2}\rangle ),\\ |{e}_{1}\rangle \to {V}_{{\rm{opt}}}|{e}_{1}\rangle  & = & \frac{1}{\sqrt{3}}(|g\rangle +\exp (-i\pi \mathrm{/6)}|{e}_{1}\rangle +\exp (-i\pi \mathrm{/3)}|{e}_{2}\rangle ),\\ |{e}_{2}\rangle \to {V}_{{\rm{opt}}}|{e}_{2}\rangle  & = & \frac{1}{\sqrt{3}}(|g\rangle +\exp (-i5\pi \mathrm{/6)}|{e}_{1}\rangle +\exp (i\pi \mathrm{/3)}|{e}_{2}\rangle \mathrm{)}.\end{array}$$

The coefficients of these three atom states differ by an amount of 2*π*/3. It implies that the optimal ladder configuration encodes the phase of 3 PSK into the three-level atom. The quantum state, which is transformed as Eq. (17), is maximally coherent^62^. It means that the optimal ladder configuration produces a maximal coherence of atomic part. Therefore, to implement the measurement strategy experimentally, the external light should encode a phase and a coherence of three-level atom state as Eq. (17) on the energy basis^63–67^.

**Figure 5:**
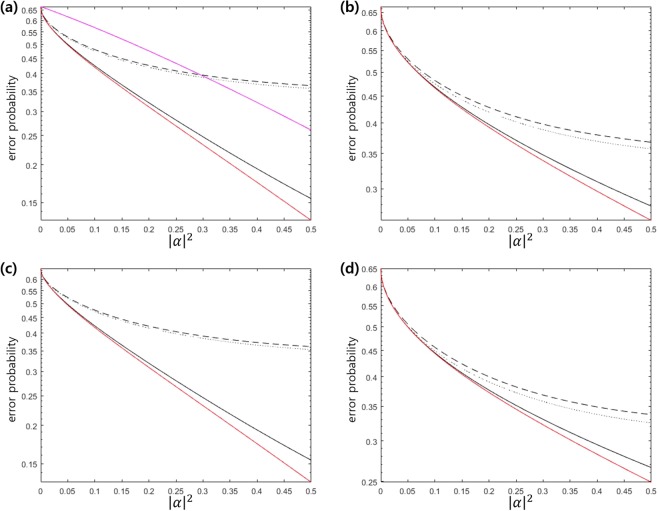
(**a**) Error probability of 3 PSK signals with identical prior probabilities (**b**) Error probability of 3 ASK signals with identical prior probabilities (**c**) Error probability of 3 PSK signals with *q*_1_ = *q*_2_ = 0.35, *q*_3_ = 0.3. (**d**) Error probability of 3 ASK signals with *q*_1_ = 0.3, *q*_2_ = *q*_3_ = 0.35. In (**a**),(**b**),(**c**), and (**d**), red solid line denotes the Helstrom bound and black dashed, dotted and solid lines are the error probability of Λ, V(*w*_1_ = 4, *w*_2_ = 1), and ladder (*w*_1_ = *w*_2_ = 1) configurations, from top to bottom. In (**a**), purple solid line is the error probability when a number of electric feedback is infinite and Dolinar-type receiver^40^ is ideal.

Ladder configuration can be used for discrimination of 3PSK with a weak amplitude. Figure 5(a) shows the error probability of 3PSK in the region of 0 ≤ |*α*|^2^ ≤ 0.5. In Fig. 5(a), Dolinar-type receiver, the cases of Λ, V, and ladder configurations are considered. The Dolinar-type receiver is the receiver proposed by S. Izumi *et al*.^40^, and the error probability is found by an infinite number of electric feedback and ideal type receiver. Figure 5(a) indicates that Λ and V configurations provide the large deviation from the Helstrom bound, but ladder configuration almost reaches the Helstrom bound. Figure 5(c) shows the error probability of 3 PSK signals at *q*_1_ = *q*_2_ = 0.35, *q*_3_ = 0.3. In this case, the Helstrom bound can be numerically obtained by semidefinite programming (SDP) problem. We find the Helstrom bound by CVX tool^68^ (The detailed analysis is given in the Method section).

The second example is the discrimination of 3ASK signals {|0〉, |*α*〉, |−*α*〉} with the identical prior probabilities. Figure 5(b) shows that ladder configuration almost reaches the Helstrom bound. However, the large deviation from the Helstrom bound can be found in Λ and V configuration as |*α*|^2^ becomes large. Figure 5(d) shows error probability of 3 ASK signals at *q*_1_ = 0.3, *q*_2_ = *q*_3_ = 0.35. In this case, the Helstrom bound can be numerically found (The detailed analysis can be found in the Method section). The gap between the Helstrom bound and the error probability obtained by Jaynes-Cummings Hamiltonian implies that there exists unextracted information^48^. The subsequent measurement of Bob may extract more information from unextracted one.

We will show that the measurement made of ladder configuration provides less error than other configurations. The ladder configuration we use is one that has the identical transition process weights. Because Λ and V configurations need two coherent lights with different polarizations, they are not suitable for minimum error discrimination of a single-shot coherent state. Also, Λ and V configurations cannot nearly reach to the Helstrom bound. Therefore, ladder configuration is better fitting for minimum error discrimination of 3 PSK or 3 ASK than Λ and V configurations.

### Quartenary coherent states discrimination

**Figure 6:**
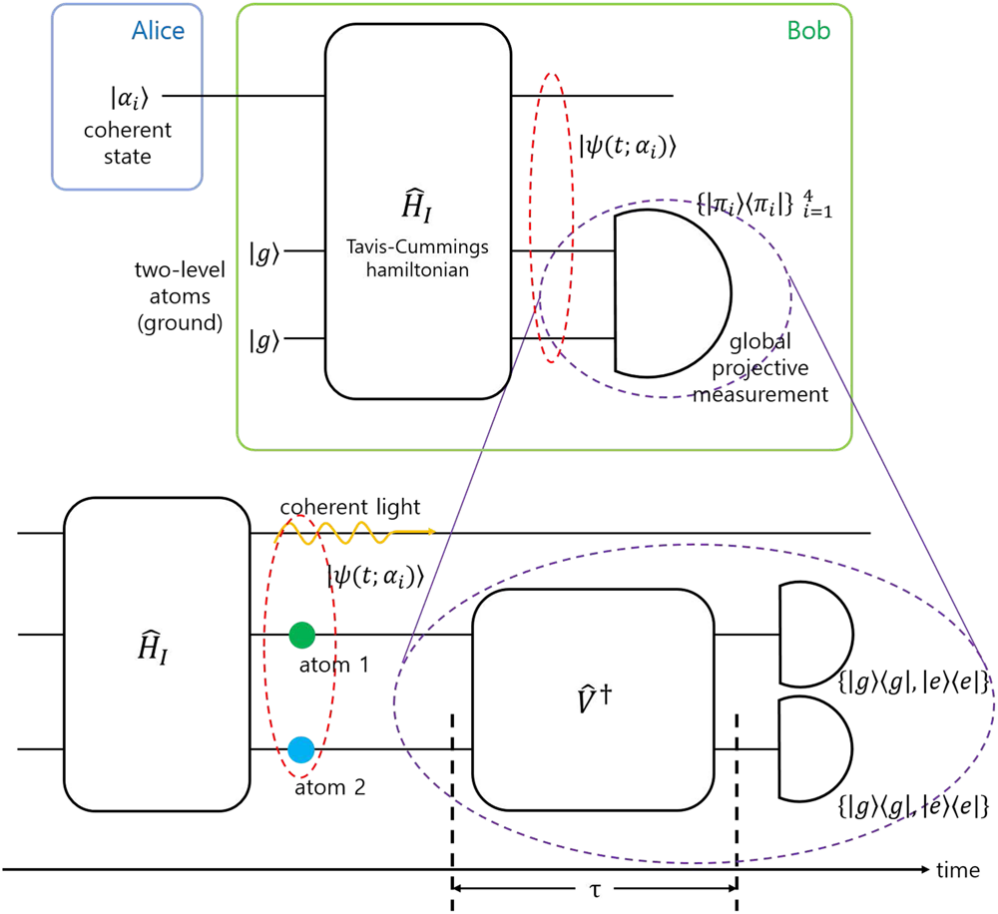
Minimum error discrimination for four coherent states of Alice. Here, Bob prepares ground state of two two-level atoms. He performs an interaction between Alice’s coherent state |*α*_*i*_〉 and two two-level atoms. Bob performs global projective measurement on quantum state of two atoms. After an interaction between a coherent state and two two-level atoms, two atoms interact with external light. After an interval of *τ*, the quantum state of two atoms is locally measured on an energy basis.

In this section, the discrimination of four coherent states is considered. Alice prepares one of four coherent states |*α*_*i*_〉 ∈ {|*α*_1_〉, |*α*_2_〉, |*α*_3_〉, |*α*_4_〉}, with equal prior probabilities. Two two-level atoms of Bob interact with the coherent state of Alice, where the interaction between the coherent state and the two two-level atoms is described by Tavis-Cummings Hamiltonian (See Fig. 6). The initial states of two two-level atoms lie in the ground states. After the interaction between the coherent state and the two two-level atoms, Bob measures the states of two two-level atoms. The guessing probability of Bob is defined by

18 $$\begin{array}{rcl}{P}_{g}=\,{\rm{\max }}\,{P}_{s} & = & \mathop{{\rm{\max }}}\limits_{{\{{\hat{\varPi }}_{i}\}}_{i=1}^{4},\Phi }\frac{1}{4}\mathop{\sum }\limits_{i=1}^{4}\,{\langle \psi (\Phi ;{\alpha }_{i}){|}_{AL}{\{|{\pi }_{i}\rangle }_{A}\langle {\pi }_{i}|\otimes {\hat{I}}_{L}\}|\psi (\Phi ;{\alpha }_{i})\rangle }_{AL}\\  & = & \mathop{{\rm{\max }}}\limits_{{\{{\hat{\varPi }}_{i}\}}_{i=1}^{3},\Phi }\frac{1}{4}\mathop{\sum }\limits_{i=1}^{4}\,|(\langle {\pi }_{i}{|}_{A}\otimes {\hat{I}}_{L})\psi (\Phi ;{\alpha }_{i}){\rangle }_{AL}{|}^{2}\mathrm{}.\end{array}$$

where, ${\{{\hat{\Pi }}_{i}\}}_{i=1}^{4}=\{|{\pi }_{i}\rangle {\langle {\pi }_{i}|\}}_{i=1}^{4}$ is a global projective measurement on two two-level atoms, which can be described by following unitary transformation

19 $$[\begin{array}{c}|{\pi }_{1}\rangle \\ |{\pi }_{2}\rangle \\ |{\pi }_{3}\rangle \\ |{\pi }_{4}\rangle \end{array}]=\tilde{V}[\begin{array}{l}|g\rangle \otimes |g\rangle \\ |g\rangle \otimes |e\rangle \\ |e\rangle \otimes |g\rangle \\ |e\rangle \otimes |e\rangle \end{array}],\,\tilde{V}=[\begin{array}{cccc}{\tilde{v}}_{11} & {\tilde{v}}_{12} & {\tilde{v}}_{13} & {\tilde{v}}_{14}\\ {\tilde{v}}_{21} & {\tilde{v}}_{22} & {\tilde{v}}_{23} & {\tilde{v}}_{24}\\ {\tilde{v}}_{31} & {\tilde{v}}_{32} & {\tilde{v}}_{33} & {\tilde{v}}_{34}\\ {\tilde{v}}_{41} & {\tilde{v}}_{42} & {\tilde{v}}_{43} & {\tilde{v}}_{44}\end{array}]\mathrm{}.$$

where $\tilde{V}$ satisfies ${\tilde{V}}^{\dagger }\tilde{V}=\tilde{V}{\tilde{V}}^{\dagger }$. Using $\tilde{V}$, the guessing probability is given by

20 $${P}_{g}=\mathop{{\rm{\max }}}\limits_{{\{{\hat{\varPi }}_{i}\}}_{i=1}^{4},\Phi }\frac{1}{4}\mathop{\sum }\limits_{i=1}^{4}\,\mathop{\sum }\limits_{n=0}^{\infty }|{\tilde{v}}_{i1}^{\ast }{c}_{g,g,n}(\Phi ;{\alpha }_{i})+{\tilde{v}}_{i2}^{\ast }{c}_{g,e,n}(\Phi ;{\alpha }_{i})+{\tilde{v}}_{i3}^{\ast }{c}_{e,g,n}(\Phi ;{\alpha }_{i})+{\tilde{v}}_{i4}^{\ast }{c}_{e,e,n}(\Phi ;{\alpha }_{i}{)|}^{2}\mathrm{}.$$

The unitary transformation $\tilde{V}$ can be represented by $\tilde{V}=\exp (i\tilde{M})$, where $\tilde{M}$ is a four dimensional hermitian matrix. By applying a non-constrained optimization method to Eq. (20), one can obtain the optimized value of Eq. (20).

**Figure 7:**
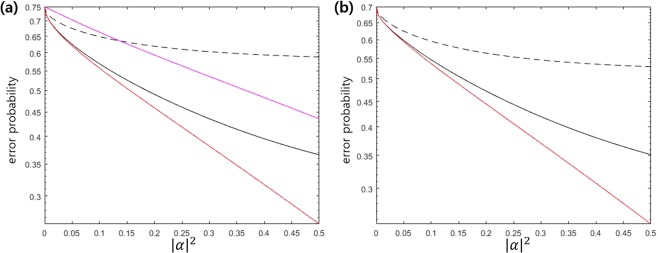
(**a**) Error probability of 4 PSK signals with identical prior probabilities (**b**) Error probability of 4 PSK signals at *q*_1_ = *q*_2_ = 0.2, *q*_3_ = *q*_4_ = 0.3. The red solid line displays the Helstrom bound. The black solid (dashed) line is the error probability of Tavis-Cummings Hamiltonian, when the unitary transformation $\tilde{V}$ is not constrained (consists of two two-dimensional unitary transformations). The purple solid line is the error probability of Dolinar-type receiver[40] without detector imperfection when the number of feedback is infinite.

As an example, let us consider 4PSK signals {|*α*〉, |*iα*〉, |−*α*〉, |−*iα*〉} with equal prior probabilities. Figure 7 shows the error probability for discrimination of 4 PSK signals when Tavis-Cummings Hamiltonian is applied. In Fig. 7, the red solid line displays the Helstrom bound. The black solid line is the error probability of Tavis-Cummings Hamiltonian, when $\tilde{V}$ is not constrained. The black dashed line is the error probability, when $\tilde{V}$ consists of two two-dimensional local unitary operators. This physically implies that the external light (*H*_*A*_ in Fig. 6) does not change entanglement between two atoms. Here, we assume that two weight *w*_1_ and *w*_2_ are identical. Figure 7 indicates that when the amplitude is small, the error probability of Tavis-Cummings Hamiltonian is smaller than that of Dolinar-type receiver^40^. However, as the amplitude becomes large, one cannot ignore the deviation from the Helstrom bound. It is because the measurement of Bob cannot extract every information of 4 PSK signals^48^ and the unextracted information still remains in the post-measurement state. Therefore, one can expect that subsequent measurement on the post-measurement state of Bob may reduce error probability.

### Revisiting ternary coherent state discrimination via symmetric tavis-cummings type interaction

Because the interaction between two-level atom and light is straightforward, instead of considering ladder configuration, Tavis-Cummings type interaction comprised of two two-level atoms can be used for discrimination of 3 coherent states.

When a symmetric coupling of Tavis-Cummings type interaction is considered, *c*_*g*,*e*,*n*−1_ and *c*_*e*,*g*,*n*−1_ in Eq. (12) are identical. Then, Eq. (12) can be expressed as follows:

21 $$\begin{array}{rcl}|\psi (t;\alpha )\rangle  & = & \mathop{\sum }\limits_{n=0}^{\infty }\,\{{c}_{g,g,n}(\Phi (t),\alpha )|g\rangle \otimes |g\rangle \otimes |n\rangle +\sqrt{2}{c}_{{\psi }_{+},n}(\Phi (t),\alpha )|{\psi }_{+}\rangle \otimes |n\rangle \\  &  & +{c}_{e,e,n}(\Phi (t),\alpha )|e\rangle \otimes |e\rangle \otimes |n\rangle \}\mathrm{}.\end{array}$$

Here, we have *c*_*ψ*+,*n*_ = *c*_*g*,*e*,*n*_ = *c*_*g*,*e*,*n*_ and $|{\psi }_{+}\rangle =(|g\rangle \otimes |e\rangle +|e\rangle \otimes |g\rangle )/\sqrt{2}$. When the symmetric coupling is assumed, $\sqrt{2}{c}_{{\psi }_{+},n}$ of Eq. (21) is the same as ${c}_{{e}_{1},n}(\sqrt{2}\Phi ;\alpha )$ of Eq. (8). When a computational basis of Eq. (14) is chosen as {|*g*〉 ⊗ |*g*〉, |*ψ*_+_〉, |*e*〉 ⊗ |*e*〉}, the minimum error discrimination of three coherent states using Tavis-Cummings type interaction is equivalent to that with ladder configuration. Here, Bob performs the atomic projective measurement of two two-level atoms. The atomic projective measurement consists of the following projector:

22 $$[\begin{array}{c}|{\pi }_{1}\rangle \\ |{\pi }_{2}\rangle \\ |{\pi }_{3}\rangle \end{array}]=V[\begin{array}{c}|g\rangle \otimes |g\rangle \\ \frac{|g\rangle \otimes |e\rangle +|e\rangle \otimes |g\rangle }{\sqrt{2}}\\ |e\rangle \otimes |e\rangle \end{array}]\mathrm{}.$$

One can assume that projective measurement comprising of Eq. (22) can be performed in the following manner. When every quantum state of two atoms is measured as the ground state, Bob guesses Alice’s coherent state as |*α*_1_〉. When every quantum state of two atoms is measured as the excited state, Bob guesses Alice’s coherent state as |*α*_3_〉. If quantum states of two atoms are measured as different quantum states, Bob guesses Alice’s coherent state as |*α*_2_〉. This measurement can be represented as a separable measurement {|*g* ⊗ *g*〉〈*g* ⊗ *g|*, |*g* ⊗ *e*〉〈*g* ⊗ *e*| + |*e* ⊗ *g*〉〈*e* ⊗ *g|*, |*e* ⊗ *e*〉〈*e* ⊗ *e*|} (|*a* ⊗ *b*〉 ≡ 〈*a* ⊗ *b*|). One can note that this separable measurement is not identical to the projective measurement of Eq. (22). In Eq. (22), the measurement element corresponding to the coherent state |*α*_2_〉 is a rank-1 projector. However, in the separable measurement, the measurement element corresponding to the coherent state |*α*_2_〉 is rank-2 projector. Therefore, the success probabilities of separable measurement and Eq. (22) are not equal to each other^69^. The reason why the measurement element corresponding to the coherent state |*α*_2_〉 in Eq. (22) is a rank-1 projector is due to entanglement.

Despite this, the success probability of the separable atomic measurement nearly reaches that of the global measurement. We compare the case of the separable atomic measurement with that of the global measurement. The guessing probability of the separable atomic measurement can be expressed as follows:

$$\begin{array}{rcl}{P}_{g} & = & \mathop{{\rm{\max }}}\limits_{{\{{\hat{\varPi }}_{i}\}}_{i=1}^{4},\Phi }\{{q}_{1}\mathop{\sum }\limits_{n=0}^{\infty }\,|{\tilde{v}}_{11}^{\ast }{c}_{g,g,n}(\Phi ;{\alpha }_{1})+{\tilde{v}}_{12}^{\ast }{c}_{g,e,,n}(\Phi ;{\alpha }_{1})+{\tilde{v}}_{13}^{\ast }{c}_{e,g,n}(\Phi ;{\alpha }_{1})+{\tilde{v}}_{14}^{\ast }{c}_{e,e,n}(\Phi ;{\alpha }_{1}{)|}^{2}\\  &  & +\,{q}_{2}\mathop{\sum }\limits_{n=0}^{\infty }\,|{\tilde{v}}_{21}^{\ast }{c}_{g,g,n}(\Phi ;{\alpha }_{2})+{\tilde{v}}_{22}^{\ast }{c}_{g,e,,n}(\Phi ;{\alpha }_{2})+{\tilde{v}}_{23}^{\ast }{c}_{e,g,n}(\Phi ;{\alpha }_{2})+{\tilde{v}}_{24}^{\ast }{c}_{e,e,n}(\Phi ;{\alpha }_{2}{)|}^{2}\\  &  & +\,{q}_{2}\mathop{\sum }\limits_{n=0}^{\infty }\,|{\tilde{v}}_{31}^{\ast }{c}_{g,g,n}(\Phi ;{\alpha }_{2})+{\tilde{v}}_{32}^{\ast }{c}_{g,e,,n}(\Phi ;{\alpha }_{2})+{\tilde{v}}_{33}^{\ast }{c}_{e,g,n}(\Phi ;{\alpha }_{2})+{\tilde{v}}_{34}^{\ast }{c}_{e,e,n}(\Phi ;{\alpha }_{2}{)|}^{2}\\  &  & +\,{q}_{3}\mathop{\sum }\limits_{n=0}^{\infty }\,|{\tilde{v}}_{41}^{\ast }{c}_{g,g,n}(\Phi ;{\alpha }_{3})+{\tilde{v}}_{42}^{\ast }{c}_{g,e,,n}(\Phi ;{\alpha }_{3})+{\tilde{v}}_{43}^{\ast }{c}_{e,g,n}(\Phi ;{\alpha }_{3})+{\tilde{v}}_{44}^{\ast }{c}_{e,e,n}(\Phi ;{\alpha }_{3}{)|}^{2}\mathrm{\}}.\end{array}$$

Here, we consider the case of *α* ∈ {0.1, 0.2, 0.3, …, 1.0}. When the prior probability of 3 PSKs is identical, the success probability of the global measurement is larger by the amount of 1.8281 × 10^−4^ than that of he separable atomic measurement. When the prior probabilities of 3 PSKs are *q*_1_ = *q*_2_ = 0.35, *q*3 = 0.3, the success probability of the global measurement is larger by the amount of 1.7443 × 10^−4^ than that of the separable atomic measurement. When the prior probabilities of 3 ASKs are *q*_1_ = 0.3, *q*_2_ = *q*_3_ = 0.35, the success probability of the global measurement is larger by the amount of 1.6580×10^−4^ than that of the separable atomic measurement.

### Information extraction by measuring atom(s)

In quantum state discrimination, the information which Bob can obtain from the quantum state of Alice can be expressed by Shannon’s mutual information between Alice and Bob. Further, the information that Bob can extract by measuring the atom can be defined as^48^

$${I}_{e}=\frac{I(A:B)}{{I}_{acc}},\,{I}_{acc}=\mathop{\max }\limits_{{\{{\varPi }_{i}\}}_{i=1}^{N}}I(A:B).$$

Here, the accessible information *I*_*acc*_ is defined as the maximized mutual information obtained by Bob’s POVM ${\{{\Pi }_{i}\}}_{i=1}^{N}$. Therefore, we have the relation 0 ≤ *I*_*e*_ ≤ 1. *I*_*e*_ is called an extracted information. When *I*_*e*_ = 1, Bob can extract all the information of Alice, by measuring the atom. However, if *I*_*e*_ < 1, Bob cannot obtain all the information of Alice, by measuring the atom. Because Bob performs a non-demolition measurement, the information that Bob cannot extract from the atom remains in the field. Therefore, the information remained in the field can be found as^48^

$${I}_{r}=1-{I}_{e},$$

where *I*_*r*_ is called residual information.

**Figure 8:**
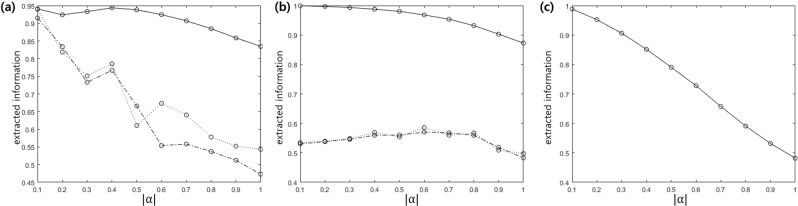
The extracted information based on atom-light interaction (**a**) 3 PSK with equal prior probabilities (**b**) 3 ASK with equal prior probabilities (**c**) 4 ASK with equal prior probabilities. In (**a**,**b**), the black solid line, the dashed line, and the dotted line show the extracted information of ladder, Λ and V configurations, respectively.

We evaluate the extracted information by maximizing a success probability. Unfortunately, except two pure qubit^48^ or particular case of pyramid state^70,71^, the analytic form of accessible information has not been known yet. In our work, we obtain the maximum of mutual information by a steepest-ascent method (The detailed analysis is given in the Method section)^72^. The extracted information is shown in Fig. 8. In Fig. 8(a–c), the quantum states of Alice are 3 PSK, 3 ASK, and 4 PSK. And the prior probability of every quantum state is identical. In Fig. 8(a,b), the black solid line shows the extracted information of ladder configuration, the dashed line displays that of Λ configuration, and the dotted line indicates that of V configuration. In Fig. 8(c) the extracted information of Tavis-Cummings interaction strategy is shown. From these, we can see that Bob obtains more information from the atom in the ladder configuration than in Λ or V configuration. This implies that the ladder configuration can reach more closely the Helstrom bound than Λ or V configuration. Also, we can see that as the amplitude |*α*| becomes large, Bob cannot extract all the information of Alice, by measuring the atom. This is the reason why the success probability cannot reach the Helstrom bound in the large |*α*|.

## Discussion

In this paper, we provided methods of minimum error discrimination for *N* coherent states, based on the idea of Han *et al*.^54^. For 3 PSK signals or 3 ASK signals with identical prior probabilities, possible interaction models can be ladder configuration, Λ configuration, or V configuration. We showed that Λ configuration and V configuration does not perform minimum error discrimination on those coherent states, but ladder configuration can provide a near value to the Helstrom bound. The extracted information of a non-demolition measurement based on the ladder configuration is larger than that of the other two configurations. Especially, the extracted information from the ladder configuration reaches nearly one. Even though the extracted information and the success probability are not proportional to each other, one may guess that as the extracted information is large, the success probability becomes large. In the case of 4 PSK signals with identical prior probabilities, we confirmed that by Tavis-Cumming model, the Helstrom bound can be nearly achieved in a region of small amplitude. The Jaynes-Cummings type interaction can be found not only in interaction between electric fields and atom but also in nuclear spin^73,74^ and superconducting qubit^75,76^. Therefore, our result can be applied to various cases. However, if the amplitude of the coherent signal becomes large, one cannot ignore the gap between the error probability and Helstrom bound. The fact becomes more noticeable as the number of coherent states becomes large. Therefore, one should find a suitable Hamiltonian for the problem. Especially to discriminate three coherent states, one should find a three-level ladder atom with *ω*_1_ = *ω*_2_. Instead of finding a three-level ladder atom, we propose that two two-level atoms may replace a ladder configuration. Meanwhile, in Λ and V configuration, the polarization of coherent state and degeneracy of atom state should be considered. Therefore, ladder configuration may be implemented easier than other configurations.

Even though we obtain the condition of unitary transformation for an atomic projective measurement, the method to experimentally implement these unitary transformations can be highly nontrivial. Therefore, further investigation on the experimental implementation of the ancilla measurements depending on the actual experimental apparatus is required. The configurations discussed in this report require two coherent lights with different frequencies or polarizations, and our work can be more useful in minimum error discrimination of two-shot coherent states. In the near future, we hope to provide a solution for minimum error discrimination of two-shot coherent states.

Even though experimental implementation should be studied further, we should note that there are two interesting research topics. The first one is to consider a scenario where multiple receivers share the information of a coherent state^25,27,29^. It is possible because our receiver does not destroy every information of the coherent state, unlike other proposed receivers^77^.

The second one is to investigate minimum error discrimination in terms of thermodynamics. It is because minimum error discrimination using a nondestructive measurement is related to the 2nd law of thermodynamics^78^. In the case of 2 PSK, when the amplitude of coherent state is sufficiently small, the nondestructive measurement performing minimum error discrimination generates a minimum of entropy. Therefore, the implementation of nondestructive minimum error discrimination in reality is meaningful. Therefore, it is interesting to analyze the relation between nondestructive minimum error discrimination and entropy generation in *N* PSK and *N* ASK.

## Methods

### Time-evolution of light-atom state

#### Ladder configuration

In this section, we analytically evaluate time evolution in the case of interaction between coherent states and three-level atom. Since the dimension of interaction Hamiltonian is infinite, we need to have a trick to solve time-dependent Schrodinger equation. Firstly, the light-atom state can be described in the following way:

23 $$\begin{array}{rcl}|\psi (t;\alpha )\rangle  & = & \mathop{\underbrace{\{{c}_{g\mathrm{,0}}(t;\alpha )|g,\,0\rangle \}}}\limits_{{\psi }_{n=0}(t;\alpha )}+\mathop{\underbrace{\{{c}_{g\mathrm{,1}}(t;\alpha )|g,\,1\rangle +{c}_{{e}_{1}\mathrm{,0}}(t;\alpha )|{e}_{1},\,0\rangle \}}}\limits_{{\psi }_{n=1}(t;\alpha )}\\  &  & +\mathop{\underbrace{\{{c}_{g\mathrm{,2}}(t;\alpha )|g,\,2\rangle +{c}_{{e}_{1}\mathrm{,1}}(t;\alpha )|{e}_{1}\mathrm{,1}\rangle +{c}_{{e}_{2}\mathrm{,0}}(t;\alpha )|{e}_{2},\,0\rangle \}}}\limits_{{\psi }_{n=2}(t;\alpha )}\\  &  & +\mathop{\underbrace{\{{c}_{g\mathrm{,3}}(t;\alpha )|g,\,3\rangle +{c}_{{e}_{1}\mathrm{,2}}(t;\alpha )|{e}_{1},\,2\rangle +{c}_{{e}_{2}\mathrm{,1}}(t;\alpha )|{e}_{2},\,1\rangle \}}}\limits_{{\psi }_{n=3}(t;\alpha )}\\  &  & +\mathop{\underbrace{\{{c}_{g\mathrm{,4}}(t;\alpha )|g,\,4\rangle +{c}_{{e}_{1}\mathrm{,3}}(t;\alpha )|{e}_{1},\,3\rangle +{c}_{{e}_{2}\mathrm{,2}}(t;\alpha )|{e}_{2},\,2\rangle \}}}\limits_{{\psi }_{n=4}(t;\alpha )}+\cdots \mathrm{}.\end{array}$$

Here, |*a*, *n*〉 = |*a*〉 ⊗ |*n*〉. In Eq. (23), {|*ψ*_*n*=0_(*t*; *α*)〉, |*ψ*_*n*=1_(*t*; *α*)〉, |*ψ*_*n*=2_(*t*; *α*)〉, …} are orthogonal each other. Therefore, interaction ${\hat{H}}_{I}^{(L)}$ can be decomposed into the following finite dimensional Hamiltonians:

24 $${\hat{H}}_{I}^{(L)}={\hat{H}}_{I,n=0}^{(L)}\oplus {\hat{H}}_{I,n=1}^{(L)}\oplus {\hat{H}}_{I,n=2}^{(L)}\oplus {\hat{H}}_{I,n=3}^{(L)}\oplus {\hat{H}}_{I,n=4}^{(L)}\oplus \cdots .$$

Here, ${\hat{H}}_{I,n=0}^{(L)}$ is a 1-dimensional Hamiltonian (real), represented by basis {|*g*〉 ⊗ |0〉}. ${\hat{H}}_{I,n=1}^{(L)}$ is a 2-dimensional Hamiltonian, given by Fock basis {|*g*〉 ⊗ |1〉, |*e*_1_〉 ⊗ |0〉}. ${\hat{H}}_{I,n\ge 2}^{(L)}$ is a 3-dimensional Hamiltonian by {|*g*〉 ⊗ |*n*〉, |*e*_1_〉 ⊗ |*n* − 1〉, |*e*_2_〉 ⊗ |*n* − 2〉}. Then, time-dependent Schrodinger equation can be written in the following way:

25 $$i\hslash \frac{\partial }{\partial t}|{\psi }_{n}\rangle ={\hat{H}}_{I,n}^{(L)}|{\psi }_{n}\rangle ,\,=0,1,2,\cdots $$

when *n* = 0, ${\hat{H}}_{I,n=0}^{(L)}$ becomes zero (real number) and we have *c*_*g*,0_ (*t*;*α)* = 0, which seems to be true. It is because the atomic state cannot be excited if there is no photon. When *n* = 1, ${\hat{H}}_{I,n=1}^{(L)}$ is identical to ref. ^50^. When *n* ≥ 2, ${\hat{H}}_{I,n}^{(L)}$ can be represented, using {|*g*〉, |*n*〉, |*e*_1_〉, |*n* − 1〉, |*e*_2_〉, |*n* − 2〉}:

26 $${\hat{H}}_{I,n}^{(L)}=\hslash g(t){\hat{M}}_{n},\,{\hat{M}}_{n}=[\begin{array}{ccc}0 & {w}_{1}^{\ast }\sqrt{n} & 0\\ {w}_{1}\sqrt{n} & 0 & {w}_{2}^{\ast }\sqrt{n-1}\\ 0 & {w}_{2}\sqrt{n-1} & 0\end{array}],\,n\ge 3.$$

The eigenvalues of hermitian matrix ${\hat{M}}_{n}$ become *λ*_1_ = *f*_*n*_,*λ*_2_ = −*f*_*n*_, and *λ*_3_ = 0, where ${f}_{n}=\sqrt{|{w}_{1}{|}^{2}n+|{w}_{2}{|}^{2}(n-\mathrm{1)}}$. The (non-normalized) eigenvector *e*_*i*_ to eigenvalue *λ*_*i*_ is given by

27 $${e}_{1,n}=[\begin{array}{c}{w}_{1}^{\ast }\sqrt{n}\\ {f}_{n}\\ {w}_{2}\sqrt{n-1}\end{array}],{e}_{2,n}=[\begin{array}{c}{w}_{1}^{\ast }\sqrt{n}\\ -{f}_{n}\\ {w}_{2}\sqrt{n-1}\end{array}],{e}_{3,n}=[\begin{array}{c}{w}_{2}^{\ast }\sqrt{n-1}\\ 0\\ -{w}_{1}\sqrt{n}\end{array}].$$

From Eq. (27), spectral decomposition of ${\hat{H}}_{I,n}$ is obtained by

28 $${\hat{M}}_{n}={\hat{U}}_{n}{\hat{\Lambda }}_{n}{\hat{U}}_{n}^{\dagger },\,{\hat{U}}_{n}=\frac{1}{\sqrt{2}{f}_{n}}[\begin{array}{ccc}{w}_{1}^{\ast }\sqrt{n} & {w}_{1}^{\ast }\sqrt{n} & {w}_{2}^{\ast }\sqrt{2(n-1)}\\ {f}_{n} & -{f}_{n} & 0\\ {w}_{2}\sqrt{n-1} & {w}_{2}\sqrt{n-1} & -{w}_{1}\sqrt{2n}\end{array}],\,{\hat{\Lambda }}_{n}=[\begin{array}{ccc}{f}_{n} & 0 & 0\\ 0 & -{f}_{n} & 0\\ 0 & 0 & 0\end{array}].$$

Then, Schrodinger equation becomes

29 $$\frac{\partial }{\partial t}\mathop{\underbrace{{\hat{U}}_{n}^{\dagger }{\psi }_{n}}}\limits_{|{\varphi }_{n}\rangle }=-ig(t){\hat{\Lambda }}_{n}\mathop{\underbrace{{\hat{U}}_{n}^{\dagger }{\psi }_{n}}}\limits_{|{\varphi }_{n}\rangle }.$$

Here, $$|{\varphi }_{n}(t)\rangle ={[\begin{array}{ccc}{v}_{1}(t) & {v}_{2}(t) & {v}_{3}(t)\end{array}]}^{{\rm{T}}}$$. Equation (29) can be analytically solved as

30 $${v}_{1}(t)={v}_{1}\mathrm{(0)}{e}^{-i{f}_{n}\varPhi (t)},\,{v}_{2}(t)={v}_{2}\mathrm{(0)}{e}^{i{f}_{n}\varPhi (t)},\,{v}_{3}(t)={v}_{3}\mathrm{(0),}\,\Phi (t)={\int }_{0}^{\infty }d\tau g(\tau \mathrm{)}.$$

From $|{\psi }_{n}\rangle ={\hat{U}}_{n}|{\varphi }_{n}\rangle$, we obtain the following relations

31 $$\begin{array}{rcl}\sqrt{2}{f}_{n}{c}_{g,n}(t) & = & {w}_{1}^{\ast }\sqrt{n}\{{v}_{1}(t)+{v}_{2}(t)\}+{w}_{2}^{\ast }\sqrt{\mathrm{2(}n-\mathrm{1)}}{v}_{3}(t),\\ \sqrt{2}{f}_{n}{c}_{{e}_{1},n-1}(t) & = & {f}_{n}\{{v}_{1}(t)-{v}_{2}(t)\},\\ \sqrt{2}{f}_{n}{c}_{{e}_{2},n-2}(t) & = & {w}_{2}\sqrt{n-1}\{{v}_{1}(t)+{v}_{2}(t)\}-{w}_{1}\sqrt{2n}{v}_{3}(t\mathrm{)}.\end{array}$$

The initial state of light-atom is |*g*〉 ⊗ |*α*〉 and we have *c*_*g*,*n*_(0) = *α*_*n*_,*c*_*e*1,*n*−1_(0) = *c*_*e*2,*n*−2_(0). Therefore, the initial condition of Eq. (31) can be found as

32 $$\begin{array}{rcl}\sqrt{2}{f}_{n}{\alpha }_{n} & = & {w}_{1}^{\ast }\sqrt{n}\{{v}_{1}\mathrm{(0)}+{v}_{2}\mathrm{(0)\}}+{w}_{2}^{\ast }\sqrt{\mathrm{2(}n-\mathrm{1)}}{v}_{3}\mathrm{(0),}\\ 0 & = & {v}_{1}(t)-{v}_{2}(t),\\ 0 & = & {w}_{2}\sqrt{n-1}\{{v}_{1}\mathrm{(0)}+{v}_{2}\mathrm{(0)\}}-{w}_{1}\sqrt{2n}{v}_{3}\mathrm{(0)}.\end{array}$$

Equation (32) is solved as

33 $${v}_{1}(0)={v}_{2}(0)=\frac{1}{\sqrt{2}{f}_{n}}{\alpha }_{n}{w}_{1}\sqrt{n},\,{v}_{3}(0)=\frac{1}{{f}_{n}}{\alpha }_{n}{w}_{2}\sqrt{n-1}.$$

Combining Eqs. (30), (31) and (33), we obtain Eq. (8). Equation (8) represents |*ψ*_*n*=0,1_〉 very well and Eq. (8) satisfies the normalization condition.

#### Λ and V configuration

In this section, we analytically evaluate time-evolution of light-atom state in Λ configuration. First of all, the light-atom state can be written as

34 $$\begin{array}{rcl}|\psi (t;\alpha )\rangle  & = & \mathop{\underbrace{{c}_{g\mathrm{,0}}(t;\alpha )|g,\,0\rangle +{c}_{{e}_{1}\mathrm{,0}}(t;\alpha )|{e}_{1},\,0\rangle }}\limits_{{\psi }_{n\mathrm{=0}}(t;\alpha )}\\  &  & +\mathop{\underbrace{{c}_{g\mathrm{,1}}(t;\alpha )|g,\,1\rangle +{c}_{{e}_{1}\mathrm{,1}}(t;\alpha )|{e}_{1},\,1\rangle +{c}_{{e}_{2}\mathrm{,0}}(t;\alpha )|{e}_{2},\,0\rangle }}\limits_{{\psi }_{n\mathrm{=1}}(t;\alpha )}\\  &  & +\mathop{\underbrace{{c}_{g\mathrm{,2}}(t;\alpha )|g,\,2\rangle +{c}_{{e}_{1}\mathrm{,2}}(t;\alpha )|{e}_{1},\,2\rangle +{c}_{{e}_{2}\mathrm{,1}}(t;\alpha )|{e}_{2},\,1\rangle }}\limits_{{\psi }_{n\mathrm{=2}}(t;\alpha )}+\cdots \mathrm{}.\end{array}$$

Here, {|*ψ*_*n*=0_(*t*; *α*)〉, |*ψ*_*n*=1_(*t*; *α*)〉, |*ψ*_*n*=2_(*t*; *α*)〉, …} are orthogonal to each other. Later, we will show *c*_*e*1,0_(*t*) = 0 for all *t*. We can decompose interaction Hamiltonian ${\hat{H}}_{I}^{(\Lambda )}$ as follows:

35 $${\hat{H}}_{I}^{(\varLambda )}={\hat{H}}_{I,n=0}^{(\varLambda )}\oplus {\hat{H}}_{I,n=1}^{(\varLambda )}\oplus {\hat{H}}_{I,n=2}^{(\varLambda )}\oplus \cdots .$$

${H}_{I,n=0}^{(\Lambda )}$ is a 2-dimensional Hamiltonian expressed by a basis of {|*g*〉 ⊗ |0〉, |*e*_1_〉 ⊗ |0〉}. ${H}_{I,n\ge 1}^{(\Lambda )}$ is a 3-dimensional Hamiltonian represented by a basis {|*g*〉 ⊗ |*n*〉, |*e*_1_〉 ⊗ |*n*〉, |*e*_2_〉 ⊗ |*n* − 1〉}. When *n* ≥ 1, ${H}_{I,n}^{(\Lambda )}$ can be written, using {|*g*〉 ⊗ |*n*〉, |*e*_1_〉 ⊗ |*n*〉, |*e*_2_〉 ⊗ |*n* − 1〉}, as follows:

36 $${\hat{H}}_{I}^{(\varLambda )}=\hslash g(t)\sqrt{n}\hat{N},\,\hat{N}=[\begin{array}{ccc}0 & 0 & {w}_{1}\\ 0 & 0 & {w}_{2}\\ {w}_{1}^{\ast } & {w}_{2}^{\ast } & 0.\end{array}]$$

The eigenvalues of hermitian matrix $\hat{N}$ are *λ*_1_ = Ω, *λ*_2_ = Ω, and ${\lambda }_{3}=\mathrm{0(}\Omega =\sqrt{|{w}_{1}{|}^{2}+|{w}_{2}{|}^{2}})$. The eigenvectors of eigenvalue *λ*_*i*_ are given by

37 $${e}_{1}=[\begin{array}{c}{w}_{1}\\ {w}_{2}\\ \varOmega \end{array}],\,{e}_{2}=[\begin{array}{c}{w}_{1}\\ {w}_{2}\\ -\varOmega \end{array}],\,{e}_{3}=[\begin{array}{c}{w}_{2}^{\ast }\\ {w}_{1}^{\ast }\\ 0\end{array}].$$

Then, the spectral decomposition of $\hat{N}$ becomes

38 $$\hat{N}=\hat{U}\hat{\Lambda }{\hat{U}}^{\dagger },\,\hat{U}=\frac{1}{\sqrt{2}\Omega }[\begin{array}{ccc}{w}_{1} & {w}_{2} & \sqrt{2}{w}_{2}^{\ast }\\ {w}_{2} & {w}_{2} & -\sqrt{2}{w}_{1}^{\ast }\\ \Omega  & -\Omega  & 0\end{array}],\,\hat{\Lambda }=[\begin{array}{ccc}\Omega  & 0 & 0\\ 0 & -\Omega  & 0\\ 0 & 0 & 0\end{array}]\mathrm{}.$$

Therefore, we can have time-dependent Schrodinger equation as follows:

39 $$\frac{\partial }{\partial t}\mathop{\underbrace{{\hat{U}}^{\dagger }|{\psi }_{n}(t)\rangle }}\limits_{|{\varphi }_{n}(t)\rangle }=-ig(t)\sqrt{n}\hat{\Lambda }\mathop{\underbrace{{\hat{U}}^{\dagger }|{\psi }_{n}(t)\rangle }}\limits_{|{\varphi }_{n}(t)\rangle },\,n\ge 1.$$

Using the expression of $$|{\varphi }_{n}(t)\rangle =[\begin{array}{ccc}{v}_{1}(t) & {v}_{2}(t) & {v}_{3}(t)\end{array}]$$, the solution of Eq. (39) can be analytically solved as follows:

40 $${v}_{1}(t)={v}_{1}\mathrm{(0)}{e}^{-i\sqrt{n}\Omega \Phi (t)},\,{v}_{2}(t)={v}_{2}\mathrm{(0)}{e}^{i\sqrt{n}\Omega \Phi (t)},\,{v}_{3}(t)={v}_{3}\mathrm{(0),}\,\Phi (t)={\int }_{0}^{t}d\tau g(\tau \mathrm{)}.$$

Applying unitary matrix $$\hat{U}$$ to $$|{\varphi }_{n}(t)\rangle $$$$(|\hat{U}{\varphi }_{n}(t)\rangle )$$, the solution of Eq. (39) can be written as

41 $$\begin{array}{rcl}{c}_{g,n}(t) & = & \frac{1}{\sqrt{2}\Omega }{w}_{1}\{{v}_{1}(t)+{v}_{2}(t)\}+\frac{1}{\Omega }{w}_{2}^{\ast }{v}_{3}(t),\\ {c}_{{e}_{1},n}(t) & = & \frac{1}{\sqrt{2}\Omega }{w}_{2}\{{v}_{1}(t)+{v}_{2}(t)\}-\frac{1}{\Omega }{w}_{1}^{\ast }{v}_{3}(t),\\ {c}_{{e}_{2},n-1}(t) & = & \frac{1}{\sqrt{2}}\{{v}_{1}(t)-{v}_{2}(t\mathrm{)\}}.\end{array}$$

Substituting an initial condition |*ψ*_*n*_(0)〉 = |*g*〉 ⊗ |*α*〉, one can obtain Eq. (9). In the similar way, one can derive Eq. (10).

### Time-evolution of light-atom state in tavis-cummings hamiltonian

In this section, we derive time-evolution of coherent state interacting with two two-level atoms. The light-atom state is expressed by

42 $$\begin{array}{rcl}|\psi (t;\alpha )\rangle  & = & \mathop{\underbrace{{c}_{g,g\mathrm{,0}}(t;\alpha )|g,\,g,\,0\rangle }}\limits_{{\psi }_{n=0}(t;\alpha )}+\mathop{\underbrace{{c}_{g,g\mathrm{,1}}(t;\alpha )|g,\,g,\,1\rangle +{c}_{g,e\mathrm{,0}}(t;\alpha )|g,\,e,\,0\rangle +{c}_{e,g\mathrm{,0}}(t;\alpha )|e,\,g,\,0\rangle }}\limits_{{\psi }_{n=1}(t;\alpha )}\\  &  & +\mathop{\underbrace{{c}_{g,g\mathrm{,2}}(t;\alpha )|g,\,g,\,2\rangle +{c}_{g,e\mathrm{,1}}(t;\alpha )|g,\,e,\,1\rangle +{c}_{e,g\mathrm{,1}}(t;\alpha )|e,\,g,\,1\rangle +{c}_{e,e\mathrm{,0}}(t;\alpha )|e,\,e,\,0\rangle }}\limits_{{\psi }_{n=2}(t;\alpha )}\\  &  & +\mathop{\underbrace{{c}_{g,g\mathrm{,3}}(t;\alpha )|g,\,g,\,3\rangle +{c}_{g,e\mathrm{,2}}(t;\alpha )|g,\,e,\,2\rangle +{c}_{e,g\mathrm{,2}}(t;\alpha )|e,\,g,\,2\rangle +{c}_{e,e\mathrm{,1}}(t;\alpha )|e,\,e,\,1\rangle }}\limits_{{\psi }_{n=3}(t;\alpha )}\cdots \mathrm{}.\end{array}$$

We use the notation of |*a*, *b*, *n*〉 = |*a*〉 ⊗ |*b*〉 ⊗ |*n*〉. {*ψ*_*n*=0_(*t*; *α*)〉, |*ψ*_*n*=1_(*t*; *α*)〉, |*ψ*_*n*=2_(*t*; *α*)〉, …} is an orthogonal set. Therefore, Tavis-Cummings Hamiltonian can be decomposed in the following manner:

43 $${\hat{H}}_{I}^{(TC)}={\hat{H}}_{I,n=0}^{(TC)}\oplus {\hat{H}}_{I,n=1}^{(TC)}\oplus {\hat{H}}_{I,n=2}^{(TC)}\oplus {\hat{H}}_{I,n=3}^{(TC)}\oplus \cdots .$$

${\hat{H}}_{I,n=0}^{(TC)}$ is a 1-dimensional Hamiltonian, expressed in terms of {|*g*, *g*, 0〉}. ${\hat{H}}_{I,n=1}^{(TC)}$ is a 3-dimensional Hamiltonian, expressed in terms of {|*g*, *g*, 1〉, |*g*, *e*, 0〉, |*e*, *g*, 0〉}. And ${\hat{H}}_{I,n\ge 2}^{(TC)}$ is a 4-dimensional Hamiltonian, expressed in terms of {|*g*, *g*, *n*〉, |*g*, *e*, *n* − 1〉, |*e*, *g*, *n* − 1〉, |*e*, *e*, *n* − 2〉}. When *n* ≥ 2, ${\hat{H}}_{I,n}^{(TC)}$ can be written by the following four-dimensional matrix:

44 $${\hat{H}}_{I,n}^{(TC)}=\hslash g(t){\hat{M}}_{n},\,{\hat{M}}_{n}=[\begin{array}{cccc}0 & \sqrt{n} & \sqrt{n} & 0\\ \sqrt{n} & 0 & 0 & \sqrt{n-1}\\ \sqrt{n} & 0 & 0 & \sqrt{n-1}\\ 0 & \sqrt{n-1} & \sqrt{n-1} & 0\end{array}].$$

The eigenvalues of Hermitian matrix ${\hat{M}}_{n}$ are ${\lambda }_{1}={\lambda }_{2}=\mathrm{0,}{\lambda }_{3}=\sqrt{\mathrm{2(2}n-\mathrm{1)}},{\lambda }_{4}=-\sqrt{\mathrm{2(2}n-\mathrm{1)}}$ and the eigenvectors to *λ*_*i*_ are given by

45 $${e}_{1}=[\begin{array}{c}0\\ -1\\ 1\\ 0\end{array}],{}_{2}=[\begin{array}{c}-\sqrt{n-1}\\ 0\\ 0\\ \sqrt{n}\end{array}],\,{e}_{3}=[\begin{array}{c}\sqrt{2n}\\ \sqrt{2n-1}\\ \sqrt{2n-1}\\ \sqrt{2(n-1)}\end{array}],\,{e}_{4}=[\begin{array}{c}\sqrt{2n}\\ -\sqrt{2n-1}\\ -\sqrt{2n-1}\\ \sqrt{2(n-1)}\end{array}].$$

Then, we obtain the following spectral decomposition of ${\hat{M}}_{n}$.

46 $$\begin{array}{ccc}{\hat{M}}_{n} & = & {\hat{U}}_{n}{\hat{\varLambda }}_{n}{\hat{U}}_{n}^{\dagger },\\ {\hat{U}}_{n} & = & [\begin{array}{cccc}0 & -\frac{\sqrt{n-1}}{\sqrt{2n-1}} & \frac{\sqrt{2n}}{2\sqrt{2n-1}} & \frac{\sqrt{2n}}{2\sqrt{2n-1}}\\ -\frac{1}{\sqrt{2}} & 0 & \frac{1}{2} & -\frac{1}{2}\\ +\frac{1}{\sqrt{2}} & 0 & \frac{1}{2} & -\frac{1}{2}\\ 0 & +\frac{\sqrt{n}}{\sqrt{2n-1}} & \frac{\sqrt{2(n-1)}}{2\sqrt{2n-1}} & \frac{\sqrt{2(n-1)}}{2\sqrt{2n-1}}\end{array}],\\ {\hat{\varLambda }}_{n} & = & [\begin{array}{cccc}0 & 0 & 0 & 0\\ 0 & 0 & 0 & 0\\ 0 & 0 & \sqrt{2(2n-1)} & 0\\ 0 & 0 & 0 & -\sqrt{2(2n-1)}\end{array}].\end{array}$$

Therefore, we have the time-dependent Schrodinger equation as follows:

47 $$\frac{\partial }{\partial t}\mathop{\underbrace{{\hat{U}}_{n}^{\dagger }{\psi }_{n}(t)}}\limits_{{\phi }_{n}(t)}=-ig(t){\hat{\varLambda }}_{n}\mathop{\underbrace{{\hat{U}}_{n}^{\dagger }{\psi }_{n}(t)}}\limits_{{\phi }_{n}(t)},\,n\ge 2.$$

Using the notation of $$|{\varphi }_{n}(t)\rangle ={[\begin{array}{cccc}{v}_{1}(t) & {v}_{2}(t) & {v}_{3}(t) & {v}_{4}(t)\end{array}]}^{{\rm{T}}}$$, one can find the solution of Eq. (47):

48 $$\begin{array}{rcl}{v}_{1}(t) & = & {v}_{1}\mathrm{(0),}\\ {v}_{2}(t) & = & {v}_{2}\mathrm{(0)},\\ {v}_{3}(t) & = & {v}_{3}\mathrm{(0)}{e}^{-i\sqrt{\mathrm{2(2}n-\mathrm{1)}}\Phi (t)},\\ {v}_{4}(t) & = & {v}_{4}\mathrm{(0)}{e}^{i\sqrt{\mathrm{2(2}n-\mathrm{1)}}\Phi (t)},\\ \Phi (t) & = & {\int }_{0}^{\infty }d\tau g(\tau \mathrm{)}.\end{array}$$

Applying unitary operator $\hat{U}$ to |*φ*_*n*_(*t*)〉, we can express the solution of Eq. (47) as follows:

$${c}_{g,g,n}(t)=-\frac{\sqrt{n-1}}{\sqrt{2n-1}}{v}_{2}(t)+\frac{\sqrt{2n}}{2\sqrt{2n-1}}\{{v}_{3}(t)+{v}_{4}(t)\},$$

49 $${c}_{g,e,n-1}(t)=-\,\frac{1}{\sqrt{2}}{v}_{1}(t)+\frac{1}{2}\{{v}_{3}(t)-{v}_{4}(t)\},$$

$${c}_{g,e,n-1}(t)=+\frac{1}{\sqrt{2}}{v}_{1}(t)+\frac{1}{2}\{{v}_{3}(t)-{v}_{4}(t)\},$$

$${c}_{e,e,n-2}(t)=\frac{\sqrt{n}}{\sqrt{2n-1}}{v}_{2}(t)+\frac{\sqrt{\mathrm{2(}n-\mathrm{1)}}}{2\sqrt{2n-1}}\{{v}_{3}(t)+{v}_{4}(t\mathrm{)\}}.$$

Substituting the initial condition |*ψ*_*n*_(0)〉 = |*α*〉 ⊗ |*g*〉 of light-atom, we obtain the follwing ones:

$${\alpha }_{n}=-\frac{\sqrt{n-1}}{\sqrt{2n-1}}{v}_{2}\mathrm{(0)}+\frac{\sqrt{2n}}{2\sqrt{2n-1}}\{{v}_{3}\mathrm{(0)}+{v}_{4}\mathrm{(0)\},}$$

50 $$0=\frac{\sqrt{n}}{\sqrt{2n-1}}{v}_{2}\mathrm{(0)}+\frac{\sqrt{2n}}{2\sqrt{2n-1}}\{{v}_{3}\mathrm{(0)}+{v}_{4}\mathrm{(0)\},}$$

$$0=-\frac{1}{\sqrt{2}}{v}_{1}\mathrm{(0)}+\frac{1}{2}\{{v}_{3}\mathrm{(0)}-{v}_{4}\mathrm{(0)\},}$$

$$0=\frac{1}{\sqrt{2}}{v}_{1}\mathrm{(0)}+\frac{1}{2}\{{v}_{3}\mathrm{(0)}-{v}_{4}\mathrm{(0)\}}.$$

*v*_*i*_(0) fulfilling Eq. (50) is given by

51 $${v}_{1}\mathrm{(0)}=\mathrm{0,}\,{v}_{2}\mathrm{(0)}=-\frac{\sqrt{n-1}}{\sqrt{\mathrm{2(2}n-\mathrm{1)}}}{\alpha }_{n},\,{v}_{3}\mathrm{(0)}={v}_{4}\mathrm{(0)}=\frac{\sqrt{n}}{\sqrt{2n-1}}{\alpha }_{n}\mathrm{}.$$

Equation (12) can be obtained by the substitution of Eqs. (48) and (51) into Eq. (49).

### Helstrom bound of 3 PSK, 4 PSK, and 3 ASK

The method to find Helstrom bound of linearly independent symmetric pure state or linearly independent partially symmetric pure state is known^1,33^. In this section, we briefly describe the method (The detailed derivation can be found in ref. ^1^).

#### Three or four psk signals case

*N* PSK has the same mathematical structure as *N* linearly independent symmetric pure states. Therefore, the minimum error discrimination of *N* PSK with identical prior probabilities can be understood by the minimum error discrimination of *N* linearly independent pure states with the following properties

52 $$|{\psi }_{k}\rangle ={V}^{k-1}|{\psi }_{1}\rangle ,\,{V}^{\dagger }V=V{V}^{\dagger }=I,\,{V}^{N}=\pm I\mathrm{}.$$

According to Kennedy’s theorem^79^, rank-1 projective measurement is the measurement for minimum error discrimination of *N* pure state (rank-1 quantum state). In addition, from the extended version of the theorem^80^, the measurement ${\{{M}_{i}\}}_{i=1}^{N}$ for minimum error discrimination of *N* quantum state ${\{{\rho }_{i}\}}_{i=1}^{N}$ satisfies the relation of rank(*M*_*i*_) ≤ rank(*ρ*_*i*_). Since coherent state is rank-1, every measurement element should be rank-1 and is orthonormal to each other. Here, let us assume that there is a following symmetry among vectors {|*w*_1_〉,…, |*w*_*n*_〉}, comprising of projective measurement:

53 $$|{w}_{k}\rangle ={V}^{k-1}|{w}_{1}\rangle \mathrm{}.$$

Then, the condition^1–4^ of minimum error discrimination to projective measurement, made of {|*w*_1_〉, …, |*w*_*n*_〉}, can be expressed by a matrix equation $G={\Omega }^{\dagger }\Omega$^1^. Here, $G={\{{\gamma }_{ij}=\langle {\psi }_{i}|{\psi }_{j}\rangle \}}_{i,j=1}^{N}$ is Gram matrix and Ω is given by $\Omega ={\{{x}_{ij}=\langle {w}_{i}|{\psi }_{j}\rangle \}}_{i,j=1}^{N}$. In order to solve the equation, let us consider the eigensystem of *G* such as *Gu*_*p*_ = *h*_*p*_*u*_*p*_, where *h*_*p*_(*u*_*p*_) is the eigenvalue of *G*(the *N*-dimensional eigenvector). Then, Helstrom bound can be expressed, in terms of *h*_*p*_, as follows^1^:

54 $${P}_{e}^{(Hel)}=1-\frac{1}{{N}^{2}}{(\mathop{\sum }\limits_{p=1}^{N}\sqrt{{h}_{p}})}^{2}\mathrm{}.$$

When *N* = 3, 4, *h*_*p*_ is given by^40^:

(N = 3)

55 $$\begin{array}{c}{h}_{1}=1+2\,\exp (-\frac{3}{2}{\alpha }^{2})\cos (\frac{\sqrt{3}}{2}{\alpha }^{2}),\\ {h}_{2}=1-\exp (-\frac{3}{2}{\alpha }^{2})\cos (\frac{\sqrt{3}}{2}{\alpha }^{2})+\sqrt{3}\exp (-\frac{3}{2}{\alpha }^{2})\sin (\frac{\sqrt{3}}{2}{\alpha }^{2}),\\ {h}_{3}=1-\exp (-\frac{3}{2}{\alpha }^{2})\cos (\frac{\sqrt{3}}{2}{\alpha }^{2})-\sqrt{3}\exp (-\frac{3}{2}{\alpha }^{2})\sin (\frac{\sqrt{3}}{2}{\alpha }^{2}),\end{array}$$

(N = 4)

56 $$\begin{array}{c}{h}_{1}=2\,\exp (\,-\,{\alpha }^{2})(\cosh \,{\alpha }^{2}+\,\cos \,{\alpha }^{2}),\\ {h}_{2}=2\,\exp (\,-\,{\alpha }^{2})(\sinh \,{\alpha }^{2}+\,\sin \,{\alpha }^{2}),\\ {h}_{3}=2\,\exp (\,-\,{\alpha }^{2})(\cosh \,{\alpha }^{2}-\,\cos \,{\alpha }^{2}),\\ {h}_{4}=2\,\exp (\,-\,{\alpha }^{2})(\sinh \,{\alpha }^{2}-\,\sin \,{\alpha }^{2}\mathrm{)}.\end{array}$$

#### Three ASK signals case

In this subsection, we explain the method to find Helstrom bound of 3 ASK signals {|0〉, |*α*〉, |−*α*〉}. Since 3 ASK signals are partially symmetric, the method is slightly different from the case of PSK signal. Gram matrix of 3 ASK is provided by

57 $$G=[\begin{array}{ccc}1 & x & {x}^{4}\\ x & 1 & {x}^{4}\\ x & {x}^{4} & 1\end{array}]\mathrm{}.$$

Here, *x* = exp(−*α*^2^/2). We have to find Ω to satisfy $G={\Omega }^{\dagger }\Omega$. Since 3 ASK are partial symmetric, one can impose partial symmetry on Ω in the following way^1^:

58 $$\varOmega =[\begin{array}{ccc}a & d & d\\ b & c & e\\ b & e & c\end{array}]\mathrm{}.$$

Then, matrix equation $G={\Omega }^{\dagger }\Omega$ can be expressed by four equations along with *ab*−*cd* = 0:

59 $$\begin{array}{c}{a}^{2}+2{b}^{2}=\mathrm{1,}\\ {d}^{2}+{c}^{2}+{e}^{2}=\mathrm{1,}\\ ad+b(c+e)=x,\\ {d}^{2}+2ce={x}^{4}\mathrm{}.\end{array}$$

*a*, *b*, *c*, *e*, satisfying the above four equations, can be solved in terms of *d*:

60 $$\begin{array}{c}a=\frac{2xd+\mathrm{(1}-{x}^{2}\mathrm{)(1}+{x}^{4}-2{d}^{2}{)}^{\mathrm{1/2}}}{1+{x}^{4}},\\ b=\frac{x{\mathrm{(1}+{x}^{4}-2{d}^{2})}^{\mathrm{1/2}}-d\mathrm{(1}-{x}^{2})}{1+{x}^{4}},\\ c=\frac{{\mathrm{(1}+{x}^{4}-2{d}^{2})}^{\mathrm{1/2}}+{\mathrm{(1}-{x}^{4})}^{\mathrm{1/2}}}{2},\\ e=\frac{{\mathrm{(1}+{x}^{4}-2{d}^{2})}^{\mathrm{1/2}}-{\mathrm{(1}-{x}^{4})}^{\mathrm{1/2}}}{2}\mathrm{}.\end{array}$$

Now, *d*, which fulfills *f*(*d*) = *ab*−*cd* = 0, can be numerically found by Newton-Raphson method. And, Helstrom bound can be obtained from ${P}_{e}^{({\rm{Hel}})}=1-({a}^{2}+2{c}^{2})/3$.

#### Other cases

The method for minimum error discrimination of more than three pure states is not known yet. Therefore, in this section, we explain the numerical method to evaluate Helstrom bound. The primal problem for minimum error discrimination is given by

61 $$\begin{array}{ll}{\rm{maximize}} & {P}_{s}=\mathop{\sum }\limits_{i\mathrm{=1}}^{N}{q}_{i}\langle {\psi }_{i}|{\hat{\Pi }}_{i}|{\psi }_{i}\rangle \\ {\rm{subject}}\,{\rm{to}} & {\hat{\Pi }}_{i}\ge 0\,\forall i\in \mathrm{\{1,}\cdots ,N\},\\  & \mathop{\sum }\limits_{i=1}^{N}{\hat{\Pi }}_{i}=\hat{I}\mathrm{}.\end{array}$$

The problem can be attacked by semidefinite programming. The duality problem of Eq. (61) is provided by^33^.

62 $$\begin{array}{ll}{\rm{minimize}} & {\rm{Tr}}\{K\}\\ {\rm{subject}}\,{\rm{to}} & K > {q}_{i}{\hat{\rho }}_{i}\,\forall i\in \mathrm{\{1,}\cdots ,N\mathrm{\}}.\end{array}$$

Here, ${\hat{\rho }}_{i}$ = |*ψ*_*i*_〉〈*ψ*_*i*_| and *K* is a Hermitian operator^4^. Equation (62) can be numerically found by CVX tool^68^. However, coherent state is given in terms of infinite dimensional bases and CVX method cannot be applied directly. Therefore, we use coherent state in the following approximate form

63 $${\alpha }_{{\rm{a}}pp}={e}^{-\frac{1}{2}|\alpha {|}^{2}}\mathop{\sum }\limits_{n=0}^{N-1}\frac{{\alpha }^{n}}{\sqrt{n!}}n\mathrm{}.$$

As *N* becomes larger, |*α*_app_〉 is close to coherent state |*α*〉. In this report, we choose *N* = 10. In this case, we can check that the numerical bounds of 3PSK, 3ASK, and 4PSK are very close to theoretical bounds of them. Matlab code can be consulted to ref. ^33^.

### Steepest-ascent method for optimizing shannon mutual information

Because Shannon mutual information between Alice and Bob is a nonlinear convex functional to Bob’s POVM ${\{{\Pi }_{i}\}}_{i=1}^{N}$, the steepest-ascent method can numerically provide the maximum of Shannon mutual information. The steepest-ascent method comprises the following three steps^72^:

Step 1. By selecting a suitable POVM {Π_*k*_}, we evaluate the Shannon mutual information $I(A:B)={\sum }_{k}{\rm{Tr}}\{{R}_{k}{\Pi }_{k}\}$.

Step 2. By choosing a proper step size *r*, we obtain ${G}_{k}=I+r({R}_{k}-{\sum }_{l}{R}_{l}{\Pi }_{l})$. Then we evaluate ${\tilde{\varPi }}_{k}={G}_{k}^{\dagger }{\Pi }_{k}{G}_{k}$.

Step 3. We find a new POVM element ${S}^{-\mathrm{1/2}}{\tilde{\Pi }}_{k}{S}^{-\mathrm{1/2}}$ ($S={\sum }_{l}{\tilde{\Pi }}_{l}$) and repeat Step 1.

Here, *R*_*k*_ is defined as follows:

$${R}_{k}=\sum _{j}{p}_{A}(j){\rho }_{j}{log}_{2}\frac{{p}_{AB}(j,k)}{{p}_{A}(j){p}_{B}(k)},\,{p}_{A}(j)={q}_{j},\,{p}_{B}(k)={\rm{Tr}}\{\mathop{\sum }\limits_{j=1}^{N}{q}_{j}{\rho }_{j}{\Pi }_{k}\}$$

By using Matlab, we perform the simulation. And the coherent state is approximated as the form of Eq. (63). If SOMIM^81^ is used, the maximum of Shannon mutual information can be obtained by the sixth degree of the decimal point.
